# Non-destructive detection of cross-sectional strain and defect structure in an individual Ag five-fold twinned nanowire by 3D electron diffraction mapping

**DOI:** 10.1038/s41598-017-06485-5

**Published:** 2017-07-24

**Authors:** Xin Fu, Jun Yuan

**Affiliations:** 1 General Research Institute for Nonferrous Metals, Beijing, 100088 P.R. China; 2 Guobiao (Beijing) Testing & Certification Co., Ltd., Beijing, 100088 P.R. China; 3 Department of Physics, University of York, York, YO10 5DD United Kingdom

## Abstract

Coherent x-ray diffraction investigations on Ag five-fold twinned nanowires (FTNWs) have drawn controversial conclusions concerning whether the intrinsic 7.35° angular gap could be compensated homogeneously through phase transformation or inhomogeneously by forming disclination strain field. In those studies, the x-ray techniques only provided an ensemble average of the structural information from all the Ag nanowires. Here, using three-dimensional (3D) electron diffraction mapping approach, we non-destructively explore the cross-sectional strain and the related strain-relief defect structures of an individual Ag FTNW with diameter about 30 nm. The quantitative analysis of the fine structure of intensity distribution combining with kinematic electron diffraction simulation confirms that for such a Ag FTNW, the intrinsic 7.35° angular deficiency results in an inhomogeneous strain field within each single crystalline segment consistent with the disclination model of stress-relief. Moreover, the five crystalline segments are found to be strained differently. Modeling analysis in combination with system energy calculation further indicates that the elastic strain energy within some crystalline segments, could be partially relieved by the creation of stacking fault layers near the twin boundaries. Our study demonstrates that 3D electron diffraction mapping is a powerful tool for the cross-sectional strain analysis of complex 1D nanostructures.

## Introduction

Strain and defects, usually coexisting in nanostructures, are known as very important structural factors influencing the mechanical^1–4^, electronic and photonic^5–7^ properties of one-dimensional (1D) nanostructures employed as building blocks for nanoscale devices. Dislocation and disclination are classic examples, and they can result in a characteristic local strain field surrounding themselves^8–10^. On the other hand, the accumulated strain energy is prone to being relieved by extended defects^11–14^. Understanding and controlling the local strain and defects in 1D nanostructures are of critical importance to improve their physical properties. However, strain state of nanostructures can be easily perturbed through sample preparation, possibly causing erroneous results. To avoid this, the non-destructive and quantitative characterization is mandatory to reveal the intrinsic strain and defects within 1D nanostructures, both longitudinally and transversely. The longitudinal lattice distortion and defect structure can be directly imaged non-destructively at atomic scale by advanced transmission electron microscopy (TEM) techniques^15–18^. However, the real-space TEM observation of transverse (cross-sectional) strain and defects in 1D nanostructures normally requires the cross-sectional sample preparation. The slicing process either by the microtome or the focused ion beam (FIB) will inevitably introduce external strain or defects interfering the realization of the intrinsic structural characteristics. Although the real-space electron tomography has detected the 3D dislocations in nanoparticle by combining 3D Fourier filter with aberration corrected high-angle annular dark-field scanning TEM (HAADF-STEM) tomography, it requires a large tilting range by using special specimen holder, and cannot be routinely used to determine the internal structures of nanomaterials, except by applying delicate and optimized reconstruction algorithm^19^.

Alternatively, comprehensive diffraction analysis in reciprocal space is also a powerful tool to detect a wealth of structural information about nanostructures. In the past decade, synchrotron-based coherent x-ray diffraction has exhibited a dramatic progress in non-destructively probing the strain field of nanoparticals^20–22^ and nanowires^23–29^. Based on the quantitative analysis of the diffraction intensity distribution, coherent x-ray diffraction has determined the axial and radial strain inside semiconductor or metallic core-shell nanowires^23, 27, 28^, as well as the cross-sectional strain within noble metal five-fold twinned nanowires^29^. Generally, x-ray diffraction only provides an average of the structural information about a bunch of nanostructures. But in the past two decades, the rapid development of coherent x-ray diffraction imaging based on oversampling method and iterative phase retrieving algorithms has enabled the identification of individual nanostructures with the currently attainable highest resolution about few nanometers^21, 30, 31^. However, the most experimental works using coherent x-ray diffraction imaging to resolve the internal strain and defects of individual nanoparticals or nanorods with high resolution have been performed on the synchrotron radiation facilities^21, 30^. The revolutions of the tabletop x-ray sources, such as x-ray free electron lasers and high harmonic generation sources, for coherent x-ray diffraction imaging are still anticipated to achieve the higher resolution the same as that by using the advanced synchrotron sources^31^. In contrast, electron diffraction based on TEM can routinely detect individual nanostructures effectively. In the scanning transmission electron microscopy (STEM) mode, nano-beam electron diffraction has been employed to map the in-plane strain of semiconductor nanostructures^32–34^. Using a 3D electron diffraction mapping approach, we have non-destructively revealed the cross-sectional twinning structure and defects of boron-rich five-fold cyclic twinned nanowires^35, 36^. To our knowledge, until now, no investigation using electron-diffraction-based techniques to identify the transverse strain field of complex 1D nanostructures has been published. In this paper, we will employ the technique of 3D electron diffraction mapping in combination with electron diffraction simulation to non-destructively detect the cross-sectional strain field and the related strain-relieving defects of Ag five-fold twinned nanowires (FTNWs).

The debate about the closure of the well-known 7.35° angular deficiency in face-centered cubic (FCC) five-fold twinned nanostructures either by homogeneous or inhomogeneous strain field has been existing for more than fifty years. In 1965, Bagley theoretically proposed homogeneous strain relaxation mechanism that the 7.35° angular deficiency could be accommodated through phase transformation from FCC to orthorhombic structure^37^. Few years later, De Wit proposed a star-disclination model suggesting that for FCC five-fold twinned structure, the twinning axis, i.e. the termination of five twin boundaries, was a partial disclination which could induce an inhomogeneous strain in five identical crystallites^9^. Experimentally, TEM investigations on the multiply twinned small particles were in favour of the existence of inhomogeneous strain field^38–40^, i.e. the star-disclination model^9^. In recent years, the internal structure identification of Ag FTNWs has attracted more attention owing to their incredible mechanical properties^4, 41, 42^, as well as fascinating application as transparent and flexible electrodes^43–45^. Coherent x-ray diffraction studies on Ag FTWNs have drawn divergent conclusions about their internal strain state. Sun *et al*. claimed to have found a novel body-centered tetragonal phase in the core of Ag FTNWs^46^, and it has recently been challenged through quantitative investigation of coherent x-ray diffraction combined with atomistic simulation that suggested the presence of star-disclination strain field instead^29^. However, due to the spatially averaging by the x-ray diffraction technique, these studies cannot detect the microstructure differences between individual Ag nanowires, or even between different single crystallites within a Ag nanowire. Here, applying the 3D electron diffraction mapping approach, we shed light on the difference of the strain state between five single crystalline segments inside an individual Ag FTNW with a diameter of 30 nm. Through the quantitative analysis of the diffracted beam intensity distribution about specific reflection reconstructed by 3D electron diffraction mapping, we confirm that the cross-sectional strain distribution in an individual Ag FTNW is inhomogeneous in accordance with the disclination model^9^, but the five single crystallites in such Ag nanowire could be differently strained. Such difference is attributed to internal strain energy relaxation by introducing variable defect microstructures. In combination with kinematic electron diffraction simulation and energetic analysis, we reveal that the partial elastic strain energy in some single crystallites could be relieved by stacking fault layers associated with partial dislocations. Our study case demonstrates that 3D electron diffraction mapping is applicable to the non-destructive determination of the transverse strain distribution and defective microstructure in complex 1D nanostructures.

## Results and Discussion

**Figure 1:**
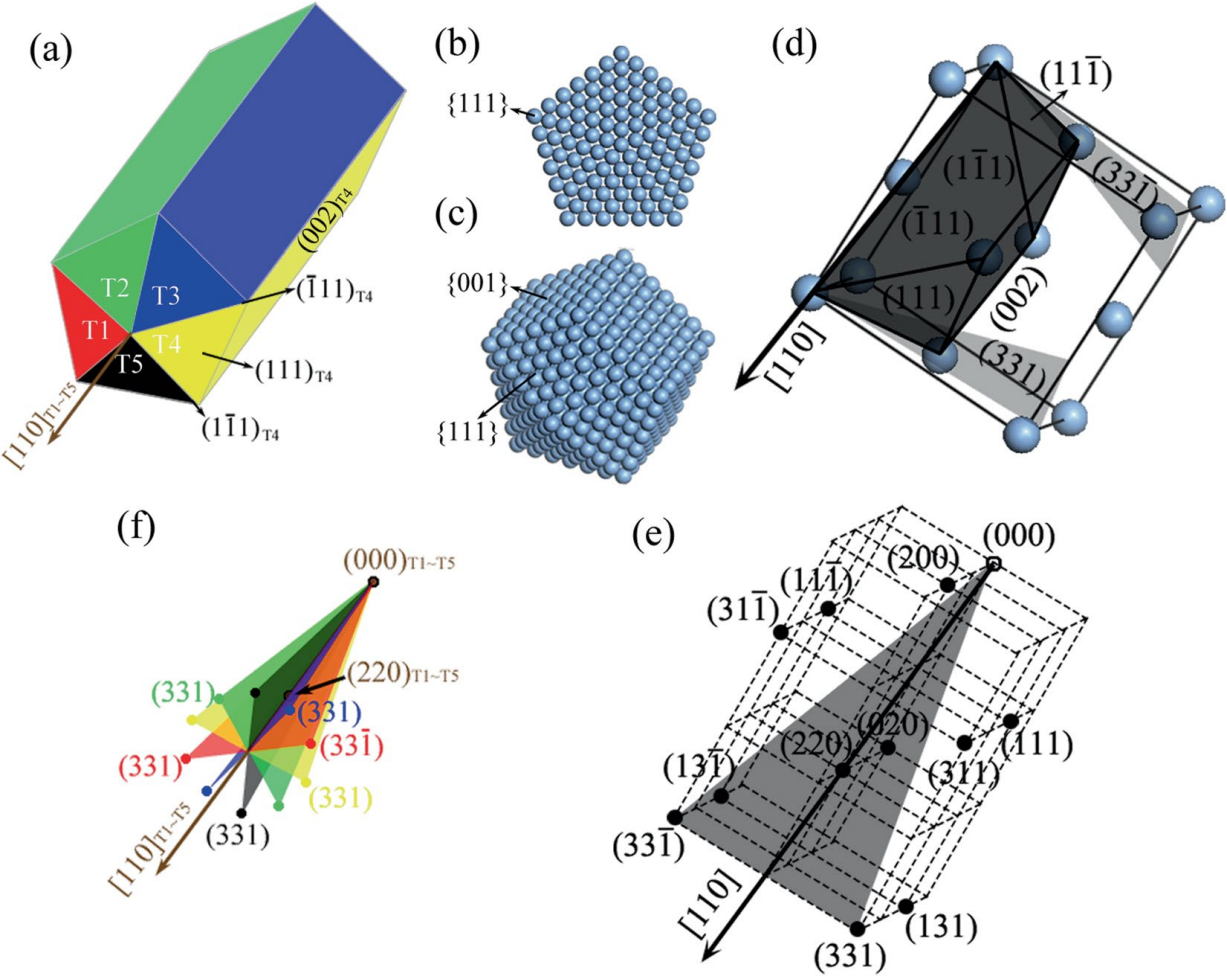
The ideal structural model and the reflection distribution in reciprocal space of Ag FTNWs. (**a**) The structural schematics of Ag FTNW. (**b**) Cross-sectional view and (**c**) 3D prospective view of the ideal atomic structural model of Ag five-fold twinned nanowires or nanoparticles. (**d**) The unit cell of Ag FCC lattice, in which the [110] direction is displayed and some specific crystallographic planes are shadowed and indexed. (**e**) The reflection distribution of a Ag FCC single crystal in the 3D reciprocal space. (**f**) 3D reciprocal-space distribution of (331) and $(33\bar{1})$ reflections near around the five-fold twinning axis, i.e. the [110] direction, shared by five single crystalline segments (T1~T5). The red, green, blue, yellow and black dots represent the reflections diffracted from T1, T2, T3, T4 and T5 segment, respectively.

The five-fold twinning structure of the Ag nanowires in this study is identified by axial-rotation electron diffraction analysis (supplementary information, Section S1, Fig. S1). Figure 1(a) demonstrates the schematic view of a Ag FTNW with five single crystalline segments labelled by T1~T5, respectively. The ideal atomic structural model for Ag FTNWs is shown in Fig. 1(b,c). Five single crystalline segments share a common [110] axis and join together at {111} twinned planes cyclically. Each segment is exposing a {001} side surface parallel to the [110] twinning axis, and capped by two {111} facets at the tips of the nanowire. To clearly view the orientation relationship of [110] twinning direction and (001) surface, as well as {111} close packing planes, we illustrate these elements in a FCC unit cell as shown in Fig. 1(d). It is obvious that each single crystalline segment for FCC Ag five-fold twinned nanostructures can be considered as a truncated tetrahedron bounded by black shadowed planes as shown in Fig. 1(d).

For a FCC single crystal, the configuration of the diffraction spots in reciprocal space is a body- centered cubic (BCC) lattice, as shown in Fig. 1(e). The [110] direction is perpendicular to (220) plane. Therefore, for the ideal Ag five-fold twinned nanostructures with [110] twinning axis, the (220) reflections from five single crystalline segments coincide in the reciprocal space. To map out a reflection group consisting of specific diffraction spots from all the single crystallites within a relative narrow tilting range, we need choose the specific reflections near around the [110] axial direction. As shown in Fig. 1(e), (331) and $(33\bar{1})$ are inversion (180° rotation) symmetric about each other around [110] axis, the dihedral angle between them is 26.53°. For the ideal structural model of Ag FTWNs as shown in Fig. 1(a), the corresponding reciprocal space distribution of the (331) and $(33\bar{1})$ reflections is demonstrated in Fig. 1(f). Each reflection pair of (331) and $(33\bar{1})$ is symmetric about [110] axis, as indicated by the colour plane, and the five reflection pairs of (331) and $(33\bar{1})$ lie in the same plane, herein called Ω for convenience. The ten diffraction peaks in the cross-sectional plane of Ω, form a regular decagonal pattern whose center is the projection of [110] twinning axis on the Ω plane. According to the dihedral angle of 26.53° between (331) and $(33\bar{1})$ plane, we can predict that the tilting range is within the allowed window from −13.3° to + 13.3°, given that the origin of the tilt angle is set at the condition that the twinning axis [110] is perpendicular to the incident beam. In the experimental operation, we choose the tilting range about from −15° to + 15° to map out all the five reflection pairs of (331) and $(33\bar{1})$.

**Figure 2:**
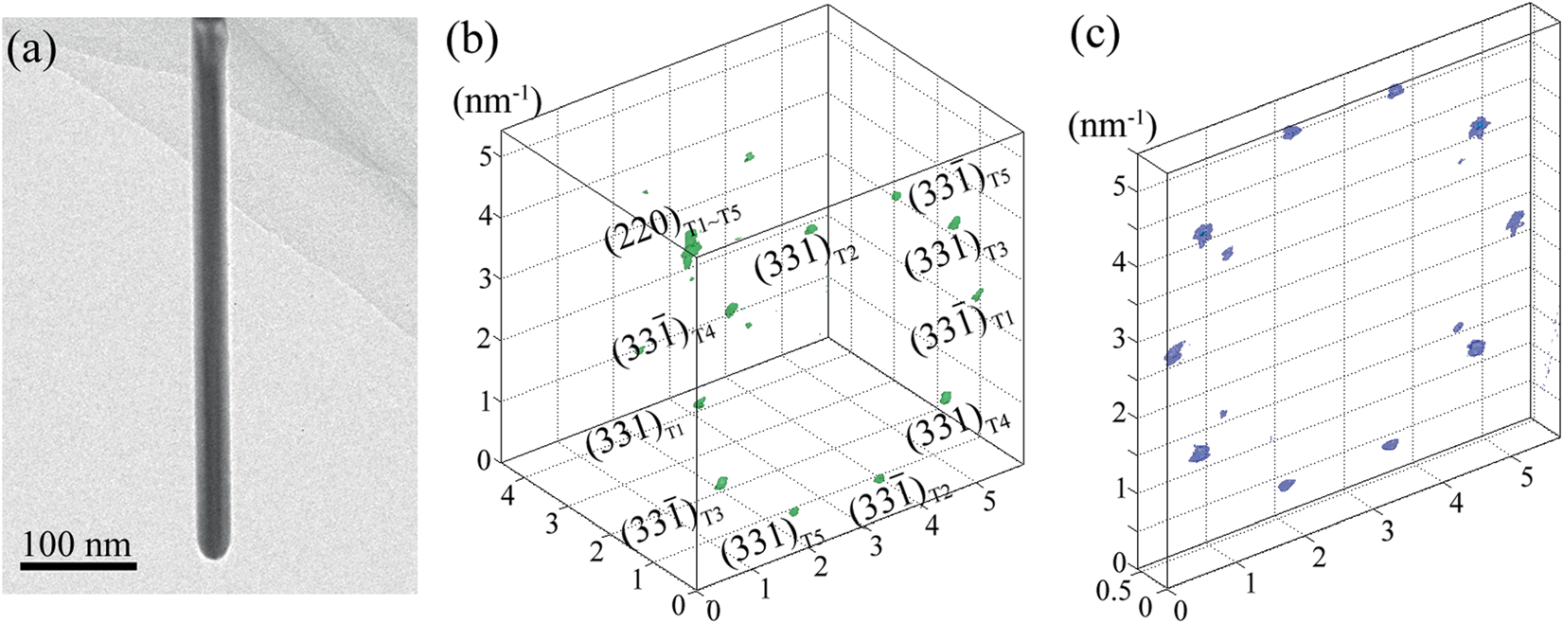
The 3D electron diffraction mapping result for a Ag FTNW. (**a**) The TEM morphology of a Ag FTNW with a diameter about 30 nm. (**b**) The 3D reconstruction result of the intensity distribution of (331), $(33\bar{1})\,$and (220) reflections from the Ag FTNW shown in (**a**). (**c**) The reciprocal volume extracted from (**b**), containing (331) and $(33\bar{1})$ reflections.

Figure 2(b) and (c) demonstrate the reconstructed reciprocal volume about the group of (331) and $(33\bar{1})$ reflection of the Ag FTNW shown in Fig. 2(a). The pentagonal pattern formed by the diffraction peaks of (331) reflection (or $(33\bar{1})$ reflection), clearly indicates the five-fold cyclic twinning structure of such Ag nanowire. As shown in Fig. 1(e) about the reciprocal lattice of a Ag single crystal, the [110] direction is defined by the reciprocal vector from the origin of the reciprocal space to the center of the line linking (331) and $(33\bar{1})$ reflection. Therefore, in Fig. 2(b), we can identify the [110] axial direction of each single crystalline segment by finding the center between (331) and $(33\bar{1})$ reflection attributable to the same crystallite. Taking the [110] axial direction of T1 segment as a reference, the axial deviation of the other four segments, T2~T5, can be evaluated. The calculated deviation angles are about 0.1°, 0.2°, 0.4° and 0.2°, respectively, and all of them are less than 0.5°. Considering the errors ascribed to the mechanical tolerance in the experimentally systematic tilting and the post alignment of the recorded diffraction patterns, it can be concluded that the five twinning crystallites of the Ag nanowire share a common long axis, i.e.[110] twinning axis.

**Figure 3:**
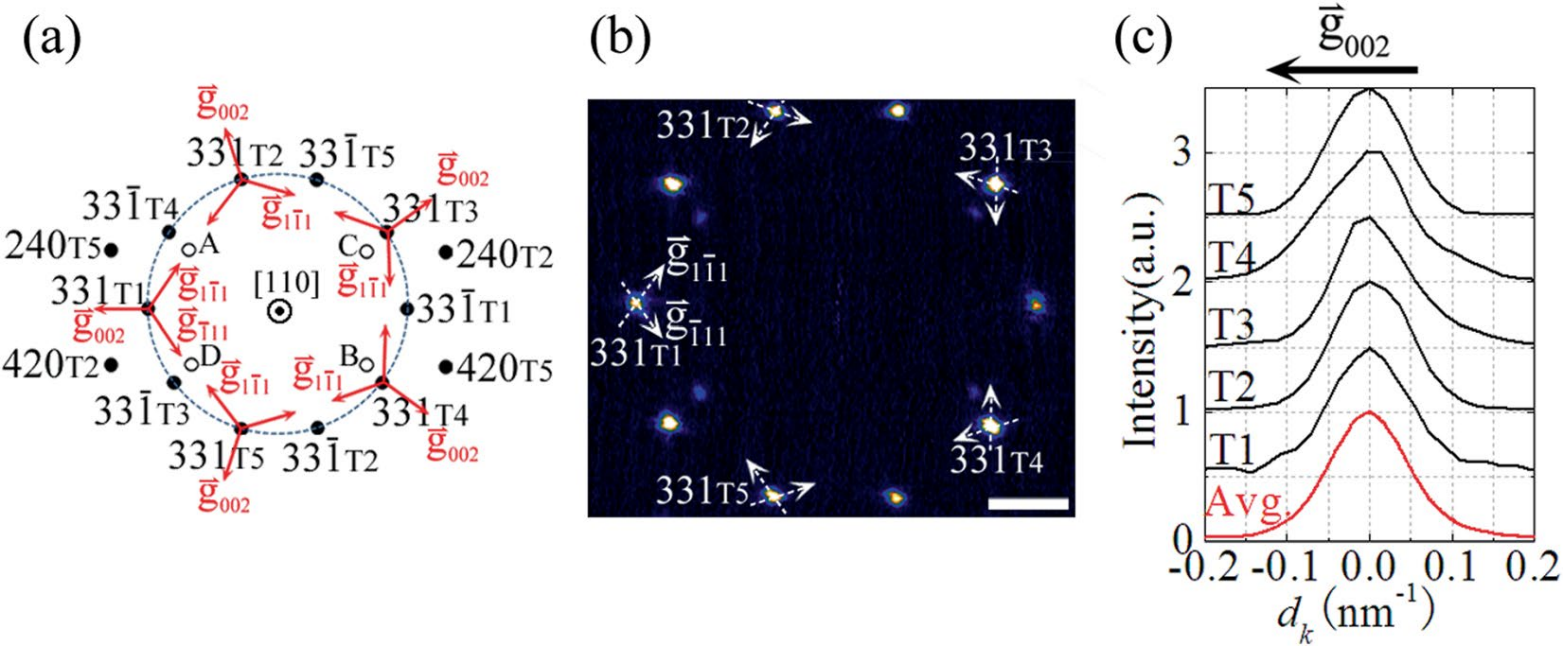
The experimental 2D intensity distribution of (331) and $(33\bar{1})$ reflection. (**a**) The pictorial demonstration drawing of the reciprocal-space cross-sectional plane of Ω, cutting through the diffraction centers of (331) and $(33\bar{1})$ reflection. The orientation of each single crystalline segment is represented using the reciprocal vectors, ${\mathop{{\rm{g}}}\limits^{ \rightharpoonup }}_{002}$, ${\mathop{{\rm{g}}}\limits^{ \rightharpoonup }}_{1\bar{1}1}$ and ${\mathop{{\rm{g}}}\limits^{ \rightharpoonup }}_{\bar{1}11}$, indicated by red arrows. The filled circles indicate the first order diffraction centers, the hollow circles represent the double reflections which are due to the (240)_T2_ (or (420)_T2_) diffracted beam rediffracted by the (002)_T1_ planes of T1 segment (the spots labelled as A and B), or attributed to it that the incident beam travelling through T1 segment is firstly diffracted by (002)_T1_ planes and then the diffracted beam passes T5 segment and is rediffracted by (240)_T5_ (or (240)_T5_) planes (the spots labelled as C and D). (**b**) The experimental 2D intensity distribution of the (331) and $(33\bar{1})$ reflections in the Ω plane. The scale bar is 1 nm^−1^. In the inner circle, the four weak spots are double reflections as schematically shown in (**a**), the labelled spots of A, B, C and D. (**c**) The intensity profiles for five single crystalline segments (T1~T5) along the direction parallel to ${\mathop{{\rm{g}}}\limits^{ \rightharpoonup }}_{002}$ vector extracted from (**b**). The five intensity curves are normalized and vertically offset. The average curve (Avg.) of the five ${\mathop{{\rm{g}}}\limits^{ \rightharpoonup }}_{002}$ experimental intensity profiles is displayed at the bottom.

**Figure 4:**
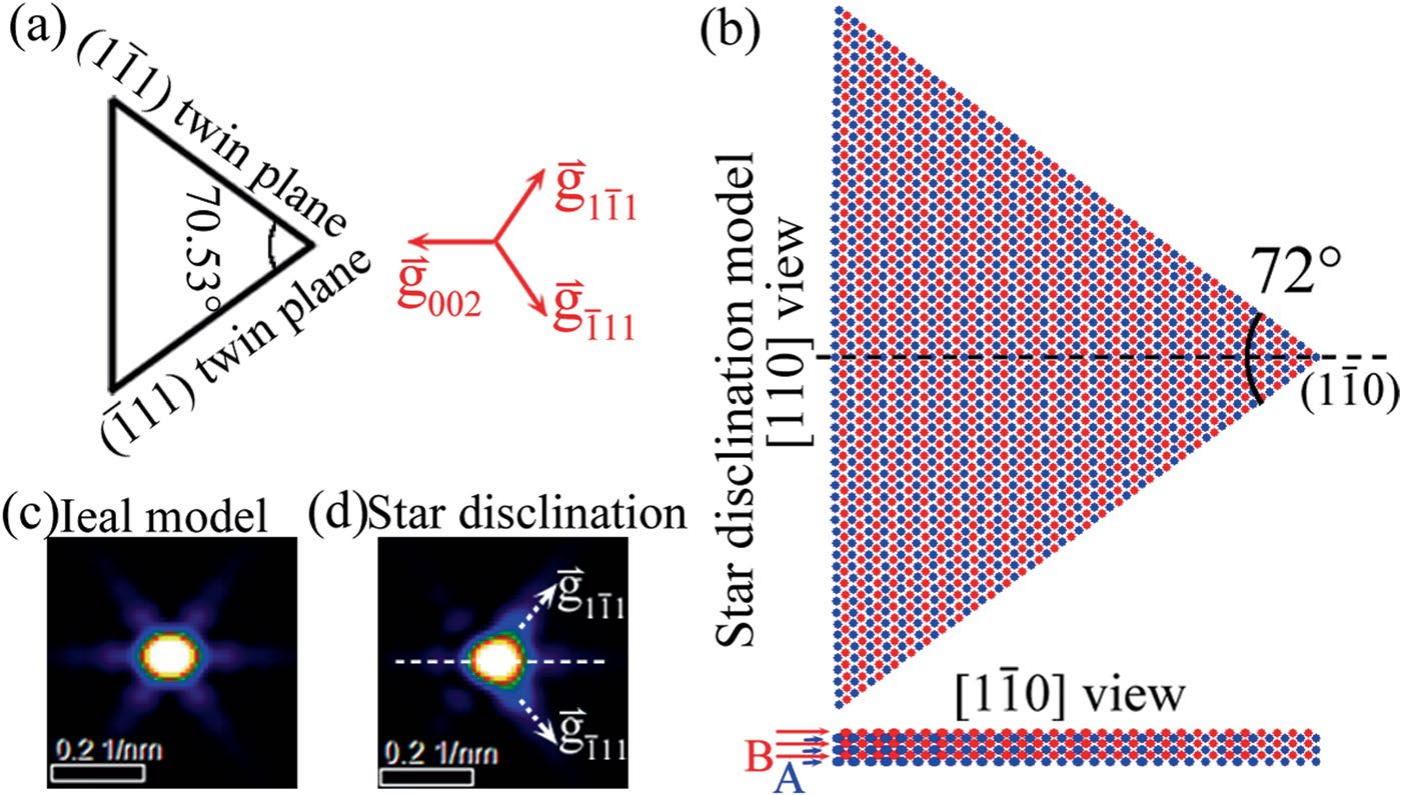
Kinematic electron diffraction simulation for the ideally unrelaxed structural model and the star-disclination model. (**a**) The schematic cross-sectional view of ideally unrelaxed structural model (left part) and the corresponding reciprocal orientation (right part). (**b**) The star-disclination atomic configuration viewing along [110] and $[1\bar{1}0]$ direction, respectively. In the $[1\bar{1}0]$ view, only four (110) layers are demonstrated. The (110) planes pack as the sequence of ABAB……. To clearly see this packing sequence in the [110] project view, the Ag atoms in A and B layers have been colored with blue and red, respectively. (**c**) and (**d**) demonstrate the kinematic electron diffraction simulation result of the 2D intensity map of (331) reflection in the Ω plane according to the ideal model (**a**) and the star-disclination model (**b**), respectively.

For nanostructures, the reciprocal-space intensity distribution in the vicinity of the diffraction centers not only reflects nanostructure’s morphology, but also contains a wealth of structural information related to the strain state and the internal defects. To further investigate the internal structure and the cross-sectional strain distribution of the Ag FTNW, the 2D intensity distribution of (331) and $(33\bar{1})$ reflections in the Ω plane has been extracted from the reconstructed 3D reciprocal volume [Fig. 2(c)] and represented in Fig. 3(b). In order to reduce the noise, the 2D intensity map shown in Fig. 3(b) is an axial integration about the reflection center with integration width of about 0.18 nm^−1^ along [110] axial direction. Considering the ideal case of the single crystalline segment which is infinitely long and without internal strain and defects [Fig. 4(a)], the (331) intensity distribution in Ω plane has been simulated by kinematic electron diffraction as shown in Fig. 4(c). The characteristic ‘flare’ seen is due to shape-induced broadening of the reflection and reflects the triangular cross-sectional morphology of the single crystalline segment as schematically shown in the left part of Fig. 4(a). In this strain-free state, the simulated 2D intensity map of (331) reflection shows an inversion (180° rotation) symmetry. However, such a symmetric feature cannot be observed in the reconstructed 2D intensity map [Fig. 3(b)] of (331) reflections diffracted by T1~T5 single crystallites of the Ag FTNW under study. We believe that such asymmetry of the experimental intensity distribution for each (331) reflection is induced by the cross-sectional strain field around the five-fold twinning axis.

First of all, we investigate the possibility of homogeneous strain relaxation through phase transformation in single crystallite. Sun *et al*. reported that a new stable phase with body-centered tetragonal (BCT) structure exists in the core of Ag FTNWs with a diameter larger than 50 nm^46^. This phase transformation is due to the internal strain caused by the five-fold twinning inside the Ag nanowires. According to the lattice structure of the new phase proposed by Sun *et al*.^46^, we calculate the reciprocal location of the (331) reflection attributable to the BCT phase. The (331) diffraction center of the BCT phase is deviated by about 0.02 nm^−1^ towards ${\mathop{{\rm{g}}}\limits^{ \rightharpoonup }}_{002}$ direction from that of the regular FCC Ag single crystallite (supplementary information, Section S2, Fig. S2). The kinematic electron diffraction simulation based on the structural model of single crystallite with BCT core and FCC sheath (the core-shell model) also indicates a shift of the (331) diffraction center towards ${\mathop{{\rm{g}}}\limits^{ \rightharpoonup }}_{002}$ direction due to the phase transformation (Supplementary information, Fig. S3). However compared with the intensity distribution [Fig. 4(c)] of the ideal structural model (pure FCC single crystalline segment as shown in Fig. 4(a)), the shape of the (331) reflection flare for the core-shell model is not significantly changed, and only the ${\mathop{{\rm{g}}}\limits^{ \rightharpoonup }}_{002}$ intensity profile shows a little asymmetry but can hardly be observed [Fig. S3]. In contrast, the shape of each (331) reflection flare in the experiment shown in Fig. 3(b) is obviously different from the simulation result of the core-shell model (supplementary information, Fig. S3). This comparison result does not support the existence of the proposed new BCT phase in the Ag FTNW.

We also extract the ${\mathop{{\rm{g}}}\limits^{ \rightharpoonup }}_{002}$ intensity profiles for the five (331) reflections from Fig. 3(b) and display them in Fig. 3(c). The (331) intensity profiles along the ${\mathop{{\rm{g}}}\limits^{ \rightharpoonup }}_{002}$ direction for T1, T2 and T5 segment are basically symmetric. Although the ${\mathop{{\rm{g}}}\limits^{ \rightharpoonup }}_{002}$ intensity profiles for T3 and T4 segments are asymmetric, their broadening is skewed in the opposite direction. The average curve in Fig. 3(c) is more symmetrical than that from individual crystallines. As the average data is closer to the x-ray emsemble average data, we can see that the details about the intrinsic inhomogeneous stress distribution between different crystallines are revealed by 3D electron diffraction mapping for the first time. This demonstrates the necessity and the advantages of resolving the strain distribution for individual over the average data.

In contrast to the homogeneous phase transformation, the inhomogeneous disclination strain field has also be proposed in Ag FTNWs to interpret the result of ensemble-averaged coherent x-ray diffraction experiment in combination with atomic simulation^29^. The well-known theoretical prediction about the inhomogeneous strain state in FCC five-fold twinned structures was the star-disclination model proposed by De Wit^9^. This model indicated that the termination of the five twinning boundaries, i.e. the twinning axis of the five-fold twinned structure, is a partial wedge disclination with five crystallites inhomogeneously strained in the same way^9^. According to De Wit’s expression^9^ about the strain field and the atomic displacement induced by the star-disclination core, we have built the atomic configuration of one single crystallite as a representative shown in Fig. 4(b) by assuming an infinitely long Ag FTNW with a diameter about 30 nm. In the $[1\bar{1}0]$ view of the established atomic configuration [Fig. 4(b)], the inhomogeneous lattice distortion can be clearly seen. The strain component analysis of this atomic configuration (supplementary information, Section S3, Fig. S4) reveals the inhomogeneous nature of the cross-sectional strain distribution. The increasing of the wedge angle between the neighboring twin planes from 70.53° to 72°, results in a lattice dilation along the $[1\bar{1}0]$ direction [Fig. S4(d)] and a compression along the [001] direction [Fig. S4(c)]. The compressive strain is most prominent near the disclination core, as well as at the corners bounded by (001) surface and {111} twin boundary. However the tensile strain is most pronounced near the center of the (001) surface. The inhomogeneous strain field totally breaks the inversion symmetry of the (331) intensity map as shown in Fig. 4(d).

Kinematic electron diffraction simulation indicates that the inhomogeneous lattice compression along the [001] direction can also induce a shift of the (331) diffraction center towards ${\mathop{{\rm{g}}}\limits^{ \rightharpoonup }}_{002}$ direction like that of the homogeneous phase transformation model, i.e. the model with BCT core and FCC sheath (Fig. S5). Although the shift distances in both of the core-shell model and the star-disclination model are of little difference in magnitude, the shape of ${\mathop{{\rm{g}}}\limits^{ \rightharpoonup }}_{002}$ intensity profiles in both models are basically all symmetric about the diffraction peaks. Thus, the direct comparison of the intensity distribution along the ${\mathop{{\rm{g}}}\limits^{ \rightharpoonup }}_{002}$ direction, cannot be used to identify whether the lattice is strained homogeneously or inhomogeneously. The simulated (331) intensity distribution in the Ω plane based on the star-disclination model is evident by streaking towards ${\mathop{{\rm{g}}}\limits^{ \rightharpoonup }}_{\bar{1}11}$ and ${\mathop{{\rm{g}}}\limits^{ \rightharpoonup }}_{1\bar{1}1}$ direction as indicated by the dashed white arrows in Fig. 4(d). Therefore, the ${\mathop{{\rm{g}}}\limits^{ \rightharpoonup }}_{\bar{1}11}$ (or ${\mathop{{\rm{g}}}\limits^{ \rightharpoonup }}_{1\bar{1}1}$) direction which is perpendicular to the twin plane can be used as the characteristic direction for the detecting of strain due to the disclination strain field.

**Figure 5:**
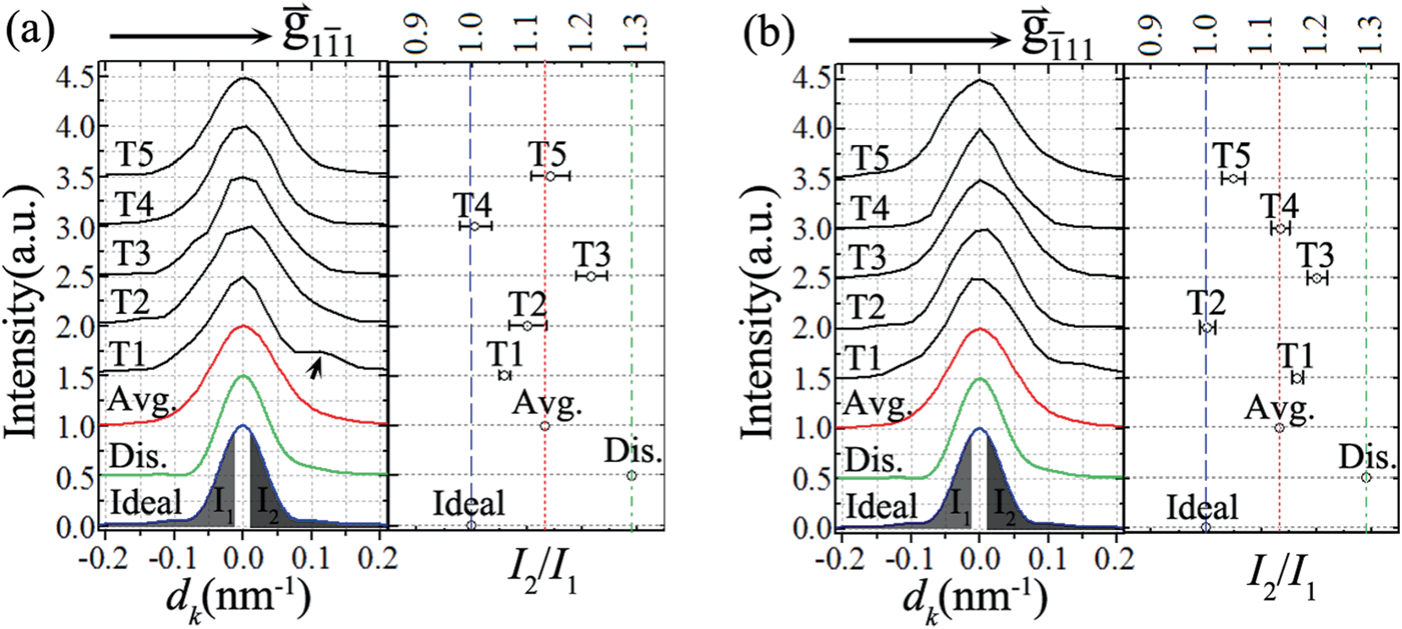
Comparison of intensity line profiles between experiment and simulation. (**a**) and (**b**) demonstrate the intensity line profile of the (331) reflection, along the direction parallel to ${\mathop{{\rm{g}}}\limits^{ \rightharpoonup }}_{1\bar{1}1}$ and ${\mathop{{\rm{g}}}\limits^{ \rightharpoonup }}_{\bar{1}11}$ vector, respectively. The experimental line profiles represented by black curves (T1~T5) are extracted from the reconstructed 2D intensity map shown in Fig. 3(b) as indicated by white dashed line with arrow. The red curve is an average (Avg.) of the ten experimental intensity profiles. The experimental intensity profiles are vertically offset and compared with the kinematically simulated result for the ideally unrelaxed structural model (Ideal, blue curve) and the star-disclination model (Dis., green curve). In the right part of (**a**) and (**b**), the ratio of the intensity integration along positive deviation (*I*_2_) to that along negative deviation (*I*_1_) is demonstrated for each intensity profile.

The similarity and the discrepancy between the experiment and the simulation based on the pure disclination model can be further identified through comparing the intensity line profiles along the characteristic directions parallel to ${\mathop{{\rm{g}}}\limits^{ \rightharpoonup }}_{1\bar{1}1}$ and ${\mathop{{\rm{g}}}\limits^{ \rightharpoonup }}_{\bar{1}11}$. The experimental intensity line profiles along ${\mathop{{\rm{g}}}\limits^{ \rightharpoonup }}_{1\bar{1}1}$ and ${\mathop{{\rm{g}}}\limits^{ \rightharpoonup }}_{\bar{1}11}$ direction, as indicated by white dashed line in Fig. 3(b), are extracted and shown in Fig. 5(a) and (b), respectively. For comparison, the experimental intensity maximum for each line profile has been normalized, and the simulated intensity profile according to the ideal model (Ideal) as well as the pure disclination model (Dis.) has been exhibit at the bottom of Fig. 5(a) and (b). For the ideal structural model, the (331) intensity profile along ${\mathop{{\rm{g}}}\limits^{ \rightharpoonup }}_{1\bar{1}1}$ or ${\mathop{{\rm{g}}}\limits^{ \rightharpoonup }}_{\bar{1}11}\,$direction shows a mirror-symmetric broadening only reflecting the finite shape effect of the cross-section morphology [Fig. 4(a)]. On the contrary, all the experimental line profiles and the simulated line profile of the pure disclination model basically show an asymmetric oscillation about the (331) diffraction peak [Fig. 5].

To quantitatively evaluate the asymmetric feature of the experimental intensity line-profile curves, we have calculated the integrated intensity ratio for each intensity curve. As displayed at the bottom in Fig. 5(a,b), herein we define the intensity integration in the deviation region from −0.01 nm^−1^ to −0.2 nm^−1^ as *I*_1_, and that in the deviation region from + 0.01 nm^−1^ to + 0.2 nm^−1^ as *I*_2_. Then the ratio of *I*_2_ to *I*_1_ for each experimental line-profile curve is calculated and shown in the right part of Fig. 5(a,b). Except for the ratio of T4 intensity profile along ${\mathop{{\rm{g}}}\limits^{ \rightharpoonup }}_{1\bar{1}1}$ direction and that of T2 intensity profile along ${\mathop{{\rm{g}}}\limits^{ \rightharpoonup }}_{\bar{1}11}$ direction approaching to the simulated ratio for the ideal case which is equal to 1 as indicated by the blue line in Fig. 5(a,b), all the other experimental integrated intensity ratios evidently exceed this simulated value. This result reveals that most of the experimental intensity line profiles in Fig. 5 show a asymmetric broadening towards the ${\mathop{{\rm{g}}}\limits^{ \rightharpoonup }}_{1\bar{1}1}$ (or ${\mathop{{\rm{g}}}\limits^{ \rightharpoonup }}_{\bar{1}11}$) direction with respect to the opposite direction. Similarly, the simulated intensity profile based on the star-disclination model (Dis. as shown in Fig. 5) also demonstrates such a feature. The similarity suggests a disclination-like inhomogeneous strain distribution in each single crystallite (T1~T5). The asymmetric feature of the intensity distribution along ${\mathop{{\rm{g}}}\limits^{ \rightharpoonup }}_{1\bar{1}1}$ (or ${\mathop{{\rm{g}}}\limits^{ \rightharpoonup }}_{\bar{1}11}$) direction possibly reflects the non-uniform distortion of the $(1\bar{1}1)$ (or $(\bar{1}11)$) atomic planes. The strain component analysis of the star-disclination model [Fig. S4] indicates that near the nanowire’s core, the atomic lattice is strongly compressed both along the ${\mathop{{\rm{g}}}\limits^{ \rightharpoonup }}_{1\bar{1}1}$ and ${\mathop{{\rm{g}}}\limits^{ \rightharpoonup }}_{\bar{1}11}$ direction, but near the $(1\bar{1}1)$ twin plane ($(\bar{1}11)$ twin plane) and the same side surface, the $(1\bar{1}1)$-type ($(\bar{1}11)$-type) atomic planes are dilated, and far from them, this type atomic planes are compressed.

Notably, there still present some differences between the experimental intensity distribution and the simulation map of the star-disclination model. Firstly, as shown in the right part of Fig. 5(a,b), for all the experimental intensity profiles along ${\mathop{{\rm{g}}}\limits^{ \rightharpoonup }}_{1\bar{1}1}$ or ${\mathop{{\rm{g}}}\limits^{ \rightharpoonup }}_{\bar{1}11}$ direction, the calculated integrated intensity ratio of *I*_2_ to *I*_1_ is less than the simulated result about 1.3 for the star-disclination model (indicated by green dot dashed line in Fig. 5). Secondly, the experimental intensity oscillation also shows a discrepancy from the simulation for the star-disclination model. As shown in Fig. 4(d), the simulated 2D intensity distribution of the star-disclination model is mirror symmetric about the middle dashed line. That is the reflection of the real-space symmetry of the atomic configuration, which is mirror symmetric about the middle $(1\bar{1}0)$ plane as shown in Fig. 4(b). Thus the simulated intensity profile along ${\mathop{{\rm{g}}}\limits^{ \rightharpoonup }}_{1\bar{1}1}$ direction is the same as that along ${\mathop{{\rm{g}}}\limits^{ \rightharpoonup }}_{\bar{1}11}$ direction [Fig. 5(a,b)]. Contrary to the pure star-disclination case, the experimental intensity distributions of the five (331) reflections do not exhibit such mirror-symmetric feature [Fig. 3(b)]. As illustrated in Fig. 5(a,b), for the single segment (T1~T5), the extracted experimental intensity profiles along ${\mathop{{\rm{g}}}\limits^{ \rightharpoonup }}_{1\bar{1}1}$ and ${\mathop{{\rm{g}}}\limits^{ \rightharpoonup }}_{\bar{1}11}$ direction show a little difference from each other. Moreover, some oscillatory features of the experimental intensity curves cannot be reproduced by the diffraction simulation based on the star-disclination model. For example, the intensity profile along ${\mathop{{\rm{g}}}\limits^{ \rightharpoonup }}_{1\bar{1}1}$ direction of T1 segment shows an evident subsidiary peak as indicated by black arrow in Fig. 5(a). But, for the star-disclintion model, the simulated intensity profile along ${\mathop{{\rm{g}}}\limits^{ \rightharpoonup }}_{1\bar{1}1}$ or ${\mathop{{\rm{g}}}\limits^{ \rightharpoonup }}_{\bar{1}11}$ direction demonstrates a continuous decay as shown in Fig. 5(a,b).

These discrepancies between theory and experiment indicate that the pure star-disclination model does not describe the internal structure of five single crystallites very well. Our reconstructed intensity map [Fig. 3(b)] and the corresponding fine structures [Figs 3(c) and 5] evidently show the difference in the intensity distribution between the five (331) reflections, which reflects the natural inhomogeneity between the five single crystallites in the individual Ag nanowire. It is worth pointing out that this structural information has not yet been detected in the published study case of Ag FTNWs with similar diameter by the coherent x-ray diffraction due to the spatial average^29^. Thus the 3D electron diffraction mapping and the coherent x-ray diffraction are complementary methods in the transverse strain analysis of complex 1D nanostructures. In Fig. 5 we also display the average curve (Avg.) of the ten experimental intensity profiles along the characteristic direction parallel to ${\mathop{{\rm{g}}}\limits^{ \rightharpoonup }}_{1\bar{1}1}$ and ${\mathop{{\rm{g}}}\limits^{ \rightharpoonup }}_{\bar{1}11}$. Obviously, the average treatment smears the fine structures originated from the individual single crystallites. The average curve shows a smooth decrease deviated from the intensity peak, like the simulated intensity profile based on the disclination model. Although both intensity curves show a similar asymmetric feature, the integrated intensity ratio (*I*_2_/*I*_1_) of the experimental average curve is still less than the simulated value for the disclination model. This further suggests the presence of defect structure involving in the internal strain relieving to compensate the intrinsic angular misfit in the real Ag FTNW under study.

Before investigating the internal structure of the Ag FTNW shown in Fig. 2(a), we should discuss about the deviation of the theoretical simulation from the real experimental condition. Firstly, the pentagonal cross-section bounded by five (001) facets used for the electron diffraction simulation is an ideal case, actually the surfaces of the real Ag FTNWs could be slightly rounded^29, 46^. Therefore, we have also built an atomic configuration of single crystalline segment with the surface rounded near the twin boundaries and the atomic lattice distorted by the star-disclination model. On the basis of this atomic configuration, the (331) diffraction intensity distribution in the Ω plane is simulated kinematically. By comparing this simulation intensity map with that simulated by the triangular cross-section model, no significant deviations can be found (supplementary information, Section S6, Fig. S7). Thus we ignore the surface morphology effect in the following discussion about the diffraction intensity fine structure. Secondly, we should consider the dynamic electron diffraction effect for the characterization of Ag FTNWs with diameters about 30 nm or more. Theoretical calculation predicted that the extinction distance for (331) reflection of FCC silver metals was about 73 nm under the condition of the incident electron beam with energy of 100 KeV^47^. Because of the inverse relationship between the extinction distance and the wave length of the incident electron beam, we guess that into the 200KeV TEM, the extinction distance for (331) reflection would be larger than 73 nm. The 30 nm diameter of the Ag nanowire under study is two times shorter than this extinction distance, indicating that the dynamic effect of the electron beam is trivial for our study. This is confirmed by the small intensity of the double diffraction peaks compared with the first order diffraction peaks in Fig. 3(b). In addition, we have also estimated the dynamic diffraction effect by assuming that the intensity of (331) diffraction beam is related with projected thickness of the Ag nanowire along the direction parallel to the incident electron beam (supplementary information, Section S7, Fig. S8). The result indicates that the experimental (331) intensity distribution in our study case has not been strongly affected by the electron multiply scattering (i.e. the dynamical diffraction effect). Therefore, the kinematic approximation is still appropriate to explain the intensity distribution of the first order electron diffraction in this study.

**Figure 6:**
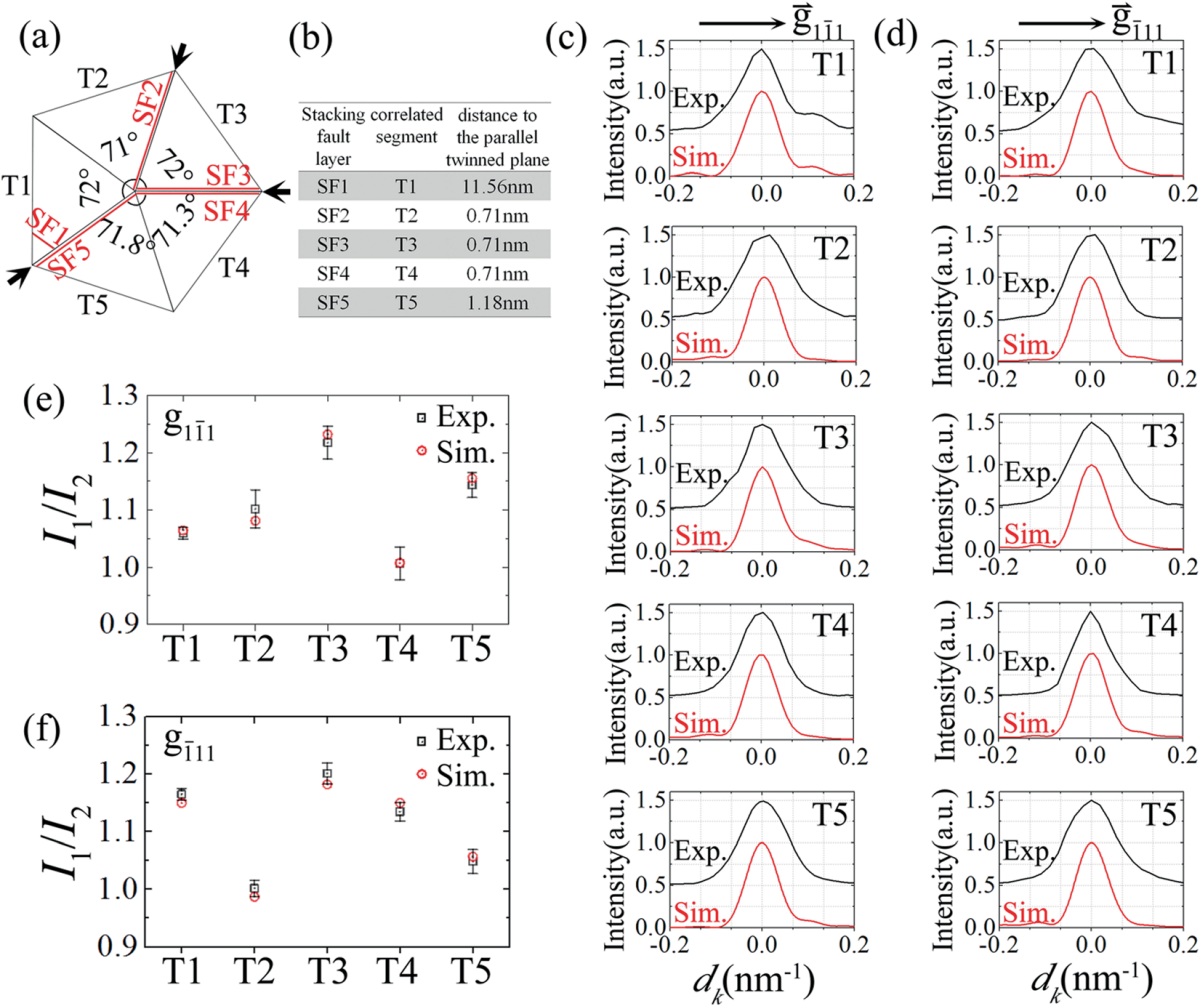
The cross-section structure model of the Ag FTNW in this study and the corresponding kinematical electron diffraction simulation. (**a**) The schematic structural model for the studied Ag FTNW. The model indicates that the inhomogeneous lattice distortion induces different angular increases of five single crystallites. Near the twinning boundaries indicated by black arrows, five stacking fault layers (SF1~SF5) are incorporated and responsible for the partial compensation of the angular misfit. The distances of five stacking fault layers to their parallel twinned plane are displayed in (**b**). According to this structural model, the kinematic electron diffraction simulation results (Sim.) for the intensity profiles of five (331) reflections along the characteristic directions parallel to ${\mathop{{\rm{g}}}\limits^{ \rightharpoonup }}_{1\bar{1}1}$ and ${\mathop{{\rm{g}}}\limits^{ \rightharpoonup }}_{\bar{1}11}$, are demonstrated in (**c**) and (**d**), respectively. In (**c**) and (**d**), the simulation intensity profiles (Sim.) are compared with their experimental counterpart (Exp.). (**e**) and (**f**) illustrate the calculated integrated intensity ratios (*I*_2_/*I*_1_ as defined in Fig. 5) of the ${\mathop{{\rm{g}}}\limits^{ \rightharpoonup }}_{1\bar{1}1}$ and ${\mathop{{\rm{g}}}\limits^{ \rightharpoonup }}_{\bar{1}11}$ line-profiles, respectively, for the five segments (T1~T5) shown in (**a**), and compare them with the corresponding experimental values.

Stacking faults and partial dislocations are usually observed in TEM microscopic analysis for FCC five-fold twinned structures^48, 49^. This is due to it that these low-energy defects are prevalent in the internal strain relieving of FCC five-fold twinned structures^13^. The star-disclination induces a continuous lattice distortion that will lead to smooth streaking of the diffraction spots. But the presence of stacking faults or micro-twins will possibly induce a more intense oscillation around the diffraction peak. To estimate the effect of stacking fault layers on the (331) intensity fine structure, we have performed a series of the kinematic electron diffraction simulation by varying the location of a single layer of stacking fault (supporting information, Section S8, Fig. S9). The simulation results indicate that for a single crystalline segment with a discliantion core in the Ag FTNW with diameter of 30 nm, the introduction of stacking fault layer in the middle of the segment (the distance of stacking fault layer to its parallel twin plane in the range from about 2 nm to 10 nm) will cause stronger subsidiary peaks, or even the diffraction spot splitting for (331) reflection along the characteristic direction of ${\mathop{{\rm{g}}}\limits^{ \rightharpoonup }}_{1\bar{1}1}$ or ${\mathop{{\rm{g}}}\limits^{ \rightharpoonup }}_{\bar{1}11}$ [Fig. S9]. In contrast, the stacking fault layer next to the twin plane or close to the corner bounded by the surface and a twin boundary can not induce obvious variation in the (331) intensity fine structure along the characteristic directions. A weak subsidiary peak possibly appears beside the (331) diffraction peak along the direction perpendicular to the stacking fault layer [Fig. S9], only in the condition that the stacking fault layer is approaching the bounded corner. Comparing the experimental result shown in Fig. 5 with the series of simulation results, we can estimate that in our experimental case, the stacking fault layers are possibly present near the twin plane or close to the corner bounded by the segment’s surface and a twin plane. Further considering the intensity fine structure of (331) reflection and the intensity integration ratio by comparing the possible theoretical structures, we propose the structural model schematically exhibited in Fig. 6(a) to interpret the fine structures of the experimental (331) intensity distribution shown in Fig. 3(b). According to this structural model, the simulated intensity ratios (*I*_2_/*I*_1_ as defined in Fig. 5) of (331) intensity profile along ${\mathop{{\rm{g}}}\limits^{ \rightharpoonup }}_{1\bar{1}1}$ and ${\mathop{{\rm{g}}}\limits^{ \rightharpoonup }}_{\bar{1}11}$ direction are basically in agreement with their experimental counterpart as demonstrated in Fig. 6(e) and (f). The kinematically simulated ${\mathop{{\rm{g}}}\limits^{ \rightharpoonup }}_{1\bar{1}1}$ and ${\mathop{{\rm{g}}}\limits^{ \rightharpoonup }}_{\bar{1}11}$ intensity profiles of (331) reflection for the five segments (T1~T5) are demonstrated in Fig. 6(c) and (d), respectively. For comparison, the normalized experimental line-profiles are also displayed and vertically offset from their simulated counterpart. It is obvious that the presence of stacking fault layers in each segment [Fig. 6(a)] breaks the mirror-symmetric feature of the pure star-disclination atomic configuration, which could explain the reciprocal-space asymmetry of the experimental intensity distribution of the five (331) reflections [Fig. 3(b)].

As shown in Fig. 6(a,b), for T1 segment, the proposed cross-sectional structure is built on the star-disclination model with wedge angle of 72° and then incorporated with a single layer of stacking fault parallel to $(1\bar{1}1)$ twin plane with distance of 11.56 nm. Based on this model, the simulated (331) intensity oscillation along the characteristic direction of ${\mathop{{\rm{g}}}\limits^{ \rightharpoonup }}_{1\bar{1}1}$ and ${\mathop{{\rm{g}}}\limits^{ \rightharpoonup }}_{\bar{1}11}$, shows a quite good agreement with the experimental (331) intensity distribution of T1 segment, both in the trend of the curve and the location of the subsidiary peak. This suggests that for the (331) experimental intensity map of T1 segment, the relatively intense flares toward ${\mathop{{\rm{g}}}\limits^{ \rightharpoonup }}_{1\bar{1}1}$ and ${\mathop{{\rm{g}}}\limits^{ \rightharpoonup }}_{\bar{1}11}$ direction, with respect to their opposite direction, are due to the inhomogeneous lattice distortion induced by the disclination core, but the subsidiary peak appearing at the intensity profile along ${\mathop{{\rm{g}}}\limits^{ \rightharpoonup }}_{1\bar{1}1}$ direction is possibly attributed to the presence of $(1\bar{1}1)$ stacking fault layer localized near the corner between the (001) surface and the $(\bar{1}11)$ twin plane as shown in Fig. 6(a). However, for the other segments, T2~T5, no significant subsidiary peaks can be detected in their intensity profiles, along the characteristic direction of ${\mathop{{\rm{g}}}\limits^{ \rightharpoonup }}_{1\bar{1}1}$ and ${\mathop{{\rm{g}}}\limits^{ \rightharpoonup }}_{\bar{1}11}$ [Fig. 6(c,d)]. This is possibly because the stacking fault layers inside T2~T5 segment near the twin boundaries with a distance of only few atomic layers cannot induce intensive oscillation [Fig. S9].

The structural model [Fig. 6(a)] also reveals two internal structural factors involving in the compensation of the 7.35° angular gap for the Ag FTNW under study. First one is the disclination core induced inhomogeneous lattice distortion inside five segments. For the ideal star-disclination model, the five segments are identical, and due to the inhomogeneous internal strain, the wedge angle between the adjacent twinned plane of each segment increases from 70.53° of the perfect FCC structure to 72°. However, for our established structural model as demonstrated in Fig. 6(a), only the wedge angles of T1 and T3 segment reach 72°, but those of T2, T4 and T5 segment are 71°, 71.3° and 71.8°, respectively, less than 72° expected for the ideal star-disclination model. The residual 1.9° angular gap could be accommodated by the imperfect twinned boundaries as indicated by black arrows in Fig. 6(a), which are incorporated with stacking faults and the accompanying partial dislocations. This indicates that part of the internal strain in the T2, T4 and T5 segment could be relieved by their internal defect structure adjacent to the twinned boundaries.

Energetically, the elastic strain energy induced by the disclination core increases exponentially with the diameter of the nanowire^9^. Thus, above a critical diameter, the introduction of extended defects with low energy could partially relieve the accumulated strain energy. Molecular dynamics simulation about Ag five-fold twinned nanorods with pentagonal cross-section has indicated a critical cross-sectional area about 450 nm^2^, beyond which the elastic strain energy is relieved by the emission of Shockley partial dislocations and the following creation of stacking fault layers^50^. In our study about the Ag FTNW with a diameter of 30 nm, the cross-sectional area is estimated to be about 589 nm^2^ [Fig. S10] larger than the critical size. Thus it is believed that there must exist internal defect structures in such Ag nanowire responsible for the elastic strain energy relieving. We have calculated the system energy of the proposed structural model shown in Fig. 6(a) and compared it with that of the corresponding pure star-dsciliantion model without defects (Methods). The calculation result indicates that the system energy is reduced by the creation of the stacking fault layers. Therefore, in an energetic view, we believe that the elastic strain energy inside Ag FTNW with a diameter of 30 nm could drive the emission of partial dislocations from the twinned plane and then further create stacking fault layers. The cross-sectional TEM observations of Ag FTNWs have shown the existence of such extended stacking faults^29, 46, 48^, however, their origins cannot be ascertained because they could easily be introduced during the preparation of the cross-section TEM sample of mechanically soft Ag nanowires. The significance of our study lies in experimental confirmation of their presence on the intact Ag nanowires.

## Conclusion

In summary, we have carried out 3D electron diffraction mapping to reconstruct the reciprocal intensity distribution of (331) reflections of a Ag five-fold twinned nanowire with a diameter about 30 nm. The geometric analysis of the relative position of (331) and $(33\bar{1})$ reflection in reciprocal space has identified the five-fold cyclic twinning structure of such Ag nanowire. There is no evidence for the presence of the strain relaxation caused by phase transformation in these nanowires with 30 nm diameter. The more intensive flare in the characteristic direction parallel to ${\mathop{{\rm{g}}}\limits^{ \rightharpoonup }}_{1\bar{1}1}$ (or ${\mathop{{\rm{g}}}\limits^{ \rightharpoonup }}_{\bar{1}11}$) vector with respect to that in the opposite direction observed in the experiment, has clearly confirmed the cross-sectional inhomogeneous strain distribution around the partial disclination core. The fine structures of the ${\mathop{{\rm{g}}}\limits^{ \rightharpoonup }}_{1\bar{1}1}$ and ${\mathop{{\rm{g}}}\limits^{ \rightharpoonup }}_{\bar{1}11}$ intensity line-profiles extracted from the five reconstructed (331) reflections have demonstrated clear evidence for uneven internal strain distribution between the five single crystalline segments and the possible existence of the strain-relieving defect structures. We have proposed an internal defect structural model combining the inhomogeneous lattice distortion induced by the partial disclination with the stacking fault layers localized near the twinned planes. The defect structural model has produced the kinematic electron diffraction simulation that is consistent with many characteristics of the experimental intensity map both in the (331) intensity fine structures and the calculated integrated intensity ratios. Energetic analysis has further suggested that this defect structural model is energetically more favourable than the pure star-disclination model. Our case study of Ag five-fold twinned nanowire also demonstrates that 3D electron diffraction intensity mapping is a powerful tool for the non-destructive identification of the transverse strain field and defect structures of complex nanostructures.

## Methods

### TEM sample preparation

The solution containing Ag FTNWs (ethanol solvent, concentration 10 mg·ml^−1^) is diluted with ethanol and then ultrasonically dispersed for about 10 minutes. The suspended solution is dropped on a referenced grid for TEM observation.

### 3D electron diffraction mapping

The 3D reconstruction of the reciprocal volume contain (331) and $(\mathrm{33}\bar{{\rm{1}}})$ reflections from the five single crystalline segments of a Ag FTNW with diameter of 30 nm is conducted through the approach of raw data recording of systematic tilting diffraction pattern and the subsequent mathematic processing of the recorded dataset. The experimental methodology and post-process of the raw dataset have be demonstrated and discussed in our previous publications^35, 36^. In this study, the systematic tilting electron diffraction mapping of the Ag nanowire is performed on a FEI Technai F20 TEM equipped with a field emission gun operated at 200 KV acceleration voltage. The tilting is conducted along the axis perpendicular to both of the nanowire’s axis and the incident electron beam, with a regular step of 0.2° and overall tilting range about from −15° to 15°, given that the tilting angle is equal to 0° at the orientation of the incident electron beam perpendicular to the nanowire’s axis. The systematic electron diffraction patterns are recorded by a Gatan 832 CCD camera. In the regular analysis by selected area electron diffraction and high-resolution TEM, Ag nanowires are easy to be deformed under the electron radiation such as bending and coiling due to the ductile nature. Thus to reduce the possible electron radiation damage for the studied Ag nanowire, experiments were conducted under low electron beam dose rate of 80 e nm^−2^ s^−1^ or less. This is a decrease by more than one order of magnitude with respect to the normal illumination condition for selected area electron diffraction.

### Kinematic electron diffraction simulation of 2D intensity map

On the kinematic approximation^51^, for an established nanoscale object only containing single type of atom with defined 3D atomic configuration, the intensity distribution around a specific reflection (*hkl*) can be given as $I\propto {|{f}_{hkl}|}^{2}{|\sum _{n}\exp (-2\pi is\cdot {r}_{n})|}^{2}$, here *s* is a reciprocal vector which defines the deviation parameter with respect to (*hkl*) diffraction center, *r*_*n*_ is a real-space vector which defines the location of each atom, and *f*_*hkl*_ represents the atomic scattering factor. In our simulation cases, on the basis of the proposed atomic configuration of Ag single crystalline segment, firstly we regularly grid a finite area in Ω plane (defined in the main text) with the origin as the (331) diffraction center, then determine the deviation parameter for each gridding element of the finite area. Thus, the 2D intensity map around (331) reflection in this finite defined area can be simulated by summing over the contribution of all the atoms in the proposed segment model for each deviation parameter in this plane. Using this approach, we carry out the kinematic electron diffraction simulation for the structural models shown in Figs 4(a,b) and 6(a), the corresponding simulation results are demonstrated in Figs 4(c,d) and 6(c,d), respectively.

### System energy calculation

The magnitude of the elastic strain energy (in an unit length) of the Ag FTNW can be estimated as GΘ^2^R^2^/16π(1−*ν*), here Θ is the characteristic rotation angle of the disclination, *ν* is Poisson’s ratio, G is the shear modulus, and R is the radius of the nanowire^9^. Currently, one reported experimental measurement of Poisson’s ratio has been carried out for Ag FTNWs with the diameter between 50 nm to 90 nm^52^. Here we can only cite the Poisson’s ratio of Ag FTNW with diameter about 50nm^52^ as an approximation for that of the Ag FTNW with diameter of 30 nm in our study case, i.e. *ν* ~ 0.225. The shear modulus is approximately evaluated according to the relationship between the shear modulus and Young’s modulus (E) for isotropic materials, G = E/2(1 + *ν*). The experimentally measured average Young’s modulus for Ag FTNW with diameter from 22 nm to 35 nm is 102 ± 23 GPa^53^, thus the shear modulus of the Ag FTNW with diameter of 30 nm is approximate to 42 GPa (G=E/2(1+v), E ~ 102 GPa, *ν* ~ 0.225). Using these values (G ~ 42 GPa, *ν* ~ 0.225), we approximately estimate the strain energy in a unit length for the pure star-dsicliantion model of Ag FTNW with diameter of 30 nm, which is about 4.0 × 10^−9^ J m^−1^ (Θ = 7.35°, for the pure star-disclination model). But for the structural model shown in Fig. 6(a), because the twinned boundaries incorporated with stacking faults and partial dislocations compensate 1.9° angular gap, the rotation angle induced by the disclination is only 5.45° corresponding to the elastic strain energy about 2.2 × 10^−9^ J m^−1^. That means the introduction of stacking faults can relieve the elastic strain energy about 1.8 × 10^−9^ J m^−1^. But we should also consider the excess energy due to the introduction of stacking faults. The earlier publication reported a experimental stacking fault energy for Ag metals^54^, which is about 22 mJ m^−2^, larger than the recent theoretical result predicted by molecular dynamics simulation^55^. Herein, using this experimental value, we estimate the excess energy due to the incorporation of stacking fault layers in our established structural model shown in Fig. 6(a). The area of the stacking fault layers in the unit length of the Ag nanowire shown in Fig. 6(a) is about 63.2 nm·m, which is estimated according the geometric relationship between the stacking fault layers and the corresponding parallel twin planes, thus the stacking fault energy in this structural model is approximately 1.4 × 10^−9^ J m^−1^, less than the reduced elastic strain energy about 1.8 × 10^−9^ J m^−1^. That means the system energy is reduced by the creation of the stacking fault layers.

## Electronic supplementary material

Supplementary Information
