# Supplementary material for: Non-destructive detection of cross-sectional strain and defect structure in an individual Ag five-fold twinned nanowire by 3D electron diffraction mapping
Source: Sci Rep. 2017 Jul 24;7:6206. doi: 10.1038/s41598-017-06485-5 (PMC5524976; doi:10.1038/s41598-017-06485-5)
Supplement: Supplementary file 1 — Supplementary Information [file 41598_2017_6485_MOESM1_ESM.pdf]

Non-destructive detection of cross-sectional strain and defect structure in an individual Ag five-fold twinned nanowire by 3D electron diffraction mapping

Supplementary Information

Xin Fu and Jun Yuan

S1. Five-fold twinned structure identification of the Ag nanowires by axial rotation electron diffraction.

Axial rotation electron diffraction has been employed to identify the internal twinning structure of the Ag nanowires in this study and clearly reveals the five-fold twinning structure in the nanowires. The twinning structure is identified as five identical face-centered cubic (FCC) single crystalline segments sharing a common [110] axis and joined at {111} twinned plane cyclically. Fig. S1 demonstrates the relative orientation relationship and the experimental results of a Ag five-fold twinned nanowire (FTNW) with diameter about 30nm identified by axial rotation electron diffraction. For convenience of expression, we have labeled the five single crystalline segments of such Ag nanowire as T1~T5, respectively.

At the 0° orientation shown in Fig. S1(a), the incident electron beam is perpendicular to the Ag nanowire's axis and the cross-section of the nanowire exhibits mirror-symmetric feature with respect to the incident electron beam. Under such a orientation relationship, the corresponding bright field image [Fig. S1(b)] and the diffraction pattern [Fig. S1(c)], also exhibit a mirror-symmetric feature. Diffraction analysis indicates that under such orientation, the electron diffraction pattern consists of $[001]_{T2}$ zone-axis reflections and $[1\bar{1}2]_{T4}$ ($[\bar{1}12]_{T5}$) zone-axis reflections as shown in Fig. S1(d).

Fig. S1(e) demonstrates another high symmetric orientation which can been obtained by rotating the Ag nanowire along its long axis by about 18° from the mirror-symmetric orientation shown in Fig. S1(a). Under such a diffraction condition, T1 is oriented at $[1\bar{1}0]$ zone-axis, T3 and T4 are oriented at $[1\bar{1}1]$ and $[\bar{1}11]$ zone-axis, respectively [Fig. S1(h)]. The reflections diffracted from T3 segment are in coincidence with that from T4 segment.

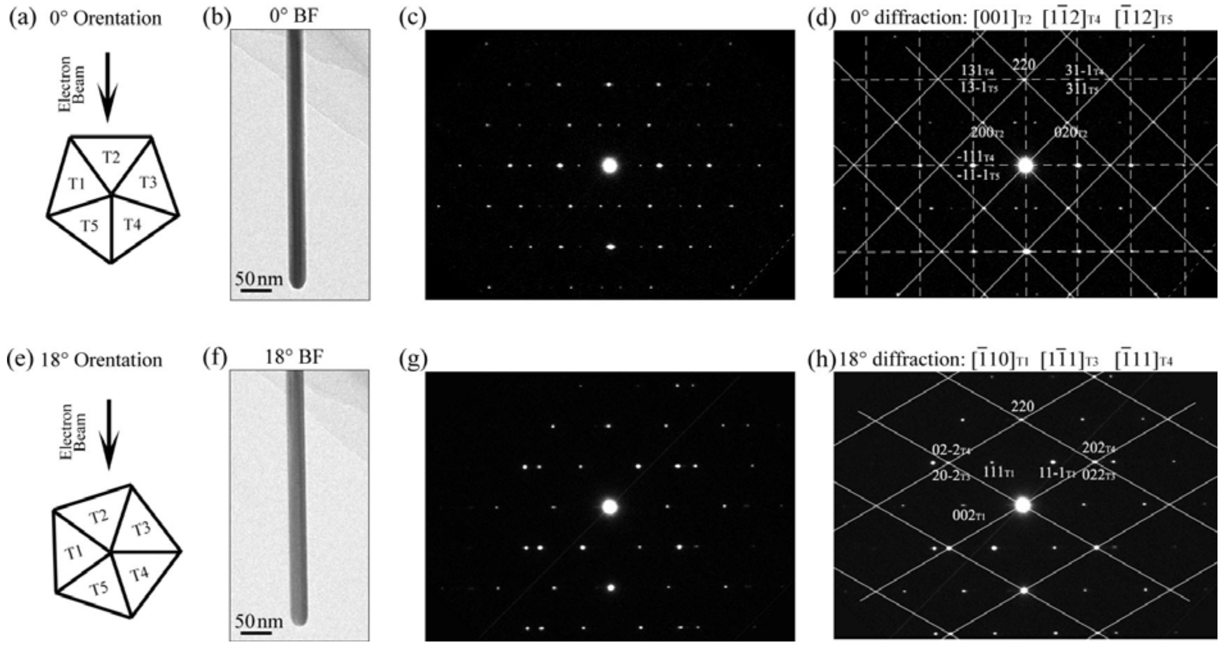

Figure S1: Five-fold twinned structure identification of a Ag nanowire under axial rotation electron diffraction condition. (a) and (e) in the first column schematically show the 0° and 18° orientation relationship, respectively. The second column, (b) and (f), as well as the third column, (c) and (g), show the corresponding bright field images and the electron diffraction patterns, respectively. In the last column, (d) and (h) illustrate the index results for the diffraction pattern shown in (c) and (g), respectively.

S2. Strain induced phase transformation

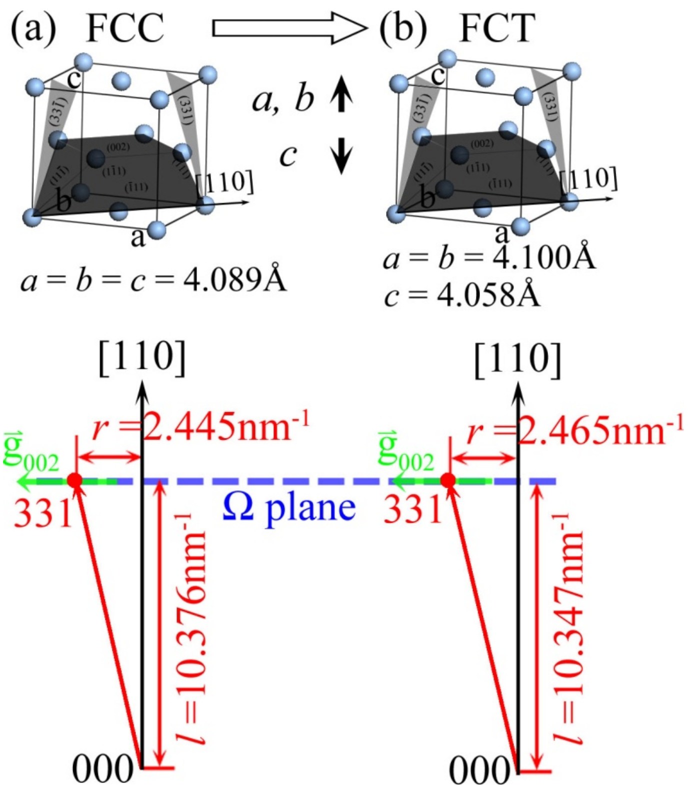

Figure S2: The deviation of (331) reflection induced by phase transformation from a FCC structure to a face-centered tetragonal (FCT) phase. (a) The upper part is a FCC unit cell. For a single crystalline segment of FCC five-fold twinned structure, the twinned planes ($(\bar{1}11)$ and $(1\bar{1}1)$ crystallographic plane), side surface ((002) crystallographic plane) and tip surfaces ((111) and $(11\bar{1})$ crystallographic plane) are indicated by black shadowed planes in the unit cell. The specific reflection planes of (331) and $(33\bar{1})$ are shadowed by gray. The lower part represents the location of (331) reflection in reciprocal space. According to the lattice parameter of Ag FCC crystal, the project distance of $\vec{g}_{331}$ vector along the nanowire's axis (i.e. [110] direction) and that on the Ω plane (defined in the main text, Fig. 3) has been calculated to be 10.376nm^{-1} and 2.445nm^{-1}, respectively. Based on Sun's prediction about the phase transformation from FCC to FCT in the core of Ag FTNWs, the lattice structure and the reciprocal location of $\vec{g}_{331}$ vector are demonstrated in the upper part and lower part, respectively.

Sun *et. al* reported that the five-fold twinning structure into Ag nanowires could induce FCC lattice distortion to form a new stable body centered tetragonal (BCT) phase in the nanowire's core.[1] The BCT phase also can be represented as a face centered tetragonal (FCT) structure with unit cell consisting of two BCT unit cells. For simplicity, here we use FCT phase to compare with the regular FCC phase as shown in Fig. S2. According to Sun's report, in the FCT unit cell, the lattice parameters of *a*, *b* and *c* are 4.100 Å, 4.100 Å, and 4.058 Å, respectively, and the direction of *c* axis is perpendicular to the nanowire's longitudinal direction, i.e. the twinning axis. Because the new phase exists in the Ag nanowires's core, we can consider that its lattice is in coincidence with that of the FCC sheath. Thus we calculate the deviation of $(331)_{FCT}$ reflection diffracted from new FCT phase from the $(331)_{FCC}$ reflection diffracted from the Ag FCC sheath as illustrated in the lower part of Fig. S2. Along the [110] direction, the $(331)_{FCT}$ diffraction center deviated by 0.03 nm^{-1} from the $(331)_{FCC}$ diffraction center. In the Ω plane the deviation of the diffraction center of $(331)_{FCT}$ reflection from that of $(331)_{FCC}$ reflection is 0.02 nm^{-1} along the direction parallel $\vec{g}_{002}$ vector.

To investigate the influence of the phase transformation on the (331) intensity distribution, we build the structural model of Ag single crystalline segment with FCT core and FCC sheath, and simulate the corresponding intensity map of (331) reflection in the Ω plane. Fig. S3(b) and (c) show two structural models with different size of the FCT core. Their corresponding (331) intensity maps are demonstrated in the right part of Fig. S3(b) and (c), respectively. The shape of the (331) reflection flares for both core-shell models do not show evident difference from that for the ideal model as shown in Fig. S3(a) (pure FCC Ag single crystalline segment). As shown in Fig. S3(d), the intensity line profiles along $\vec{g}_{1\bar{1}1}$ (or $\vec{g}_{\bar{1}11}$) direction of the tow core-shell models and the pure FCC ideal model are basically shown a symmetric feature. The detectable difference can be found in the $\vec{g}_{002}$ intensity profiles. As shown in Fig. S3(e), the phase transformation of the segment core would induce a shift of (331) diffraction center

towards $\vec{g}_{002}$ direction, this is in agreement with the geometric analysis result shown in Fig. S2. Additionally, the intensity profiles of the core-shell models show a little asymmetry [Fig. S3(f)], but it is hardly observed in the 2D intensity map as shown in Fig. S3(b) and (c). The comparison results indicate that the phase transformation in the segment core would not significantly influence the intensity distribution of (331) reflection.

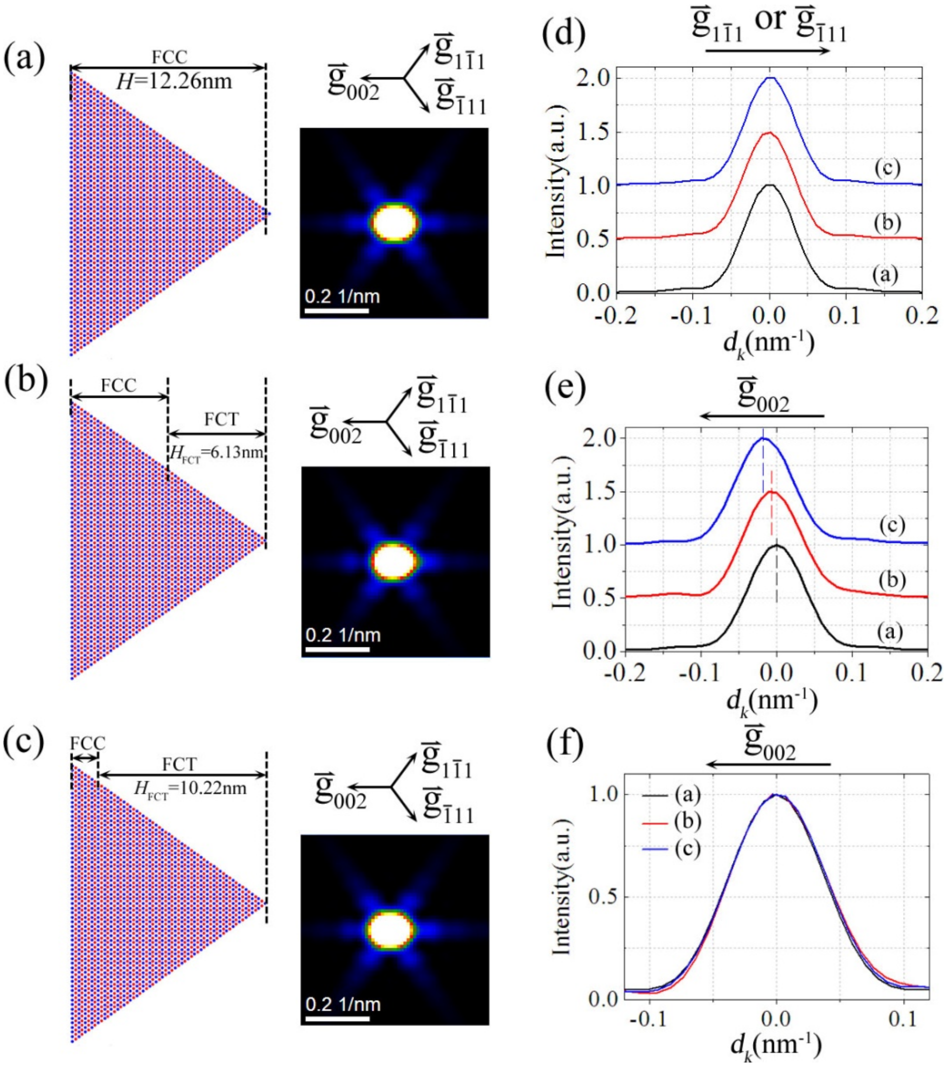

Figure S3: The structural models about single crystallite with FCT core and FCC sheath. (a) The left part is the cross-sectional atomic configuration of the ideal model without lattice distortion, i.e. pure FCC Ag crystalline segment. The corresponding simulated (331) intensity distribution is shown in the right part. (b) and (c) demonstrate the atomic configurations of two models about single crystallite with FCT core and FCC sheath, their corresponding (331) intensity distributions simulated by the kinematic approximation are also exhibit in the right

part, respectively. (d) and (e) compare the extracted intensity profiles along $\vec{g}_{1\bar{1}1}$ (or $\vec{g}_{\bar{1}11}$) and $\vec{g}_{002}$ direction, respectively, for the three structural models shown in (a), (b) and (c). In (e), the origin of the deviation (d_k) is set at the diffraction center of (331) reflection for pure FCC Ag crystalline segment shown in (a). To compare the curve shape of the $\vec{g}_{002}$ intensity profiles extracted from the intensity map shown in (a), (b), and (c), respectively, the three profiles are displayed together in (f) by aligning their intensity maximum at the same origin.

S3.Star-disclination strain field

Assuming an infinitely long isotropic cylinder with a wedge disclination axis, the disclination core induces a strain field perpendicular to the disclination axis, the corresponding atomic displacement in cylindrical coordinates, *r* and ϕ, can be expressed as followings. [2]

$$u_r = \frac{\Theta}{4\pi} r \left\{ \frac{1-2\nu}{1-\nu} \ln\left(\frac{r}{R}\right) - 1 \right\} \qquad (1)$$

$$u_\phi = \frac{\Theta}{2\pi} r\phi \qquad (2)$$

Here, *R* is the radius of the cylinder, Θ is the characteristic rotation angle of the disclination, and ν is Poisson's ratio. For FCC silver five-fold twinned structure, the rotation angle Θ is 7.35°. For the Poisson's ratio of Ag FTWN with diameter of 30nm we use the experimentally measured Poisson's ratio of Ag FTNW with diameter about 50nm as an approximate reference, i.e. ν~0.225.[3]

According to De Wit's expression [2] about the strain field and the atomic displacement induced by the star-disclination core as mentioned above, we have built the atomic configuration of one single crystallite as a representative shown in Fig. S4(b) by assuming an infinitely long Ag FTNW with diameter about 30nm. On the basis of the established star-dsiclination atomic configuration, the corresponding cross-sectional strain distribution has

also been calculated. As shown in the left upper corner of Fig. S4(a), using T1 as the representative segment, we have defined the Cartesian coordinate system with X and Y direction parallel to the [001] and $[1\bar{1}0]$ crystallographic direction, respectively. According to this Cartesian coordinate system, the color maps of the cross-sectional strain component η_{xx}, η_{yy} and η_{xy}, are superimposed on the atomic configuration, as illustrated in Fig. S4(c), (d) and (e), respectively. The inhomogeneous nature of the strain distribution can be clearly seen from the strain component maps. These maps indicate that the increasing of the wedge angle between the neighboring twin planes from 70.53° to 72°, results in a lattice dilation along $[1\bar{1}0]$ direction [Fig. S4(d)] and a compression along [001] direction [Fig. S4(c)]. As shown in Fig. S4 (c), the compressive strain is most prominent near the disclination core, as well as at the corners bounded by (001) surface and {111} twin boundary. However the tensile strain as illustrated in Fig. S4(d) is most pronounced near the center of the (001) surface. The shear strain, η_{xy}, is also non-uniform, it is gradually increasing from the segment middle plane (indicated by the black dashed line) towards the twin boundaries as indicated by the black arrows in Fig. S4(e).

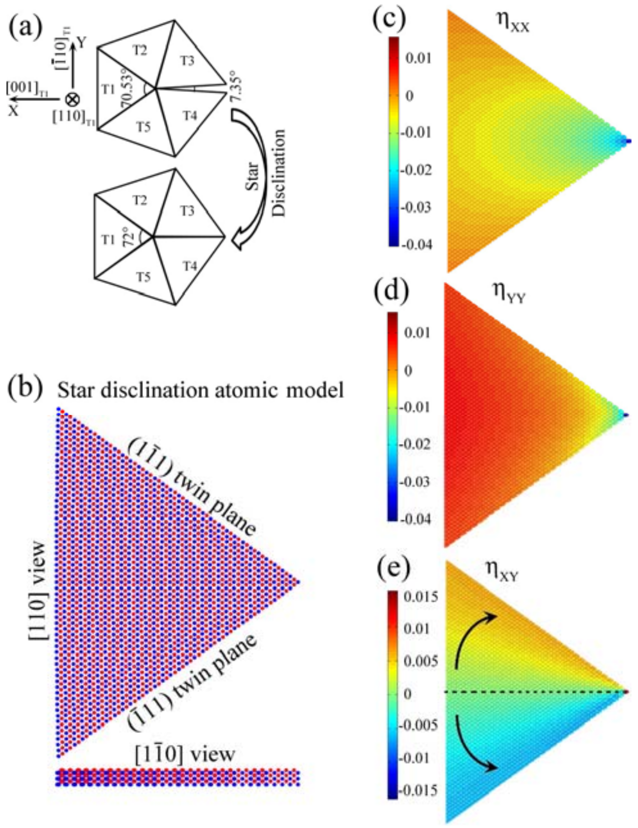

Figure S4: Star-disclination model and the simulated (331) intensity distribution map. (a) The cross-sectional schematic view of the star-disclination model with the 7.35° angular deficiency accommodated through inhomogeneous strain field. The Cartesian coordinate system and the corresponding crystallographic directions for T1 segment of the Ag FTNW are also demonstrated. (b) The atomic configuration of the star-disclination model applied to a Ag FTNW with diameter of 30nm. Only one segment with the same orientation as T1 demonstrated in (a) is shown. (c), (d) and (e) is the color map of the strain component, η_{xx}, η_{yy} and η_{xy}, respectively, with the Cartesian coordinate system defined in (a), calculated according to the atomic configuration shown in (b).

S4. Strain induced diffraction center shift

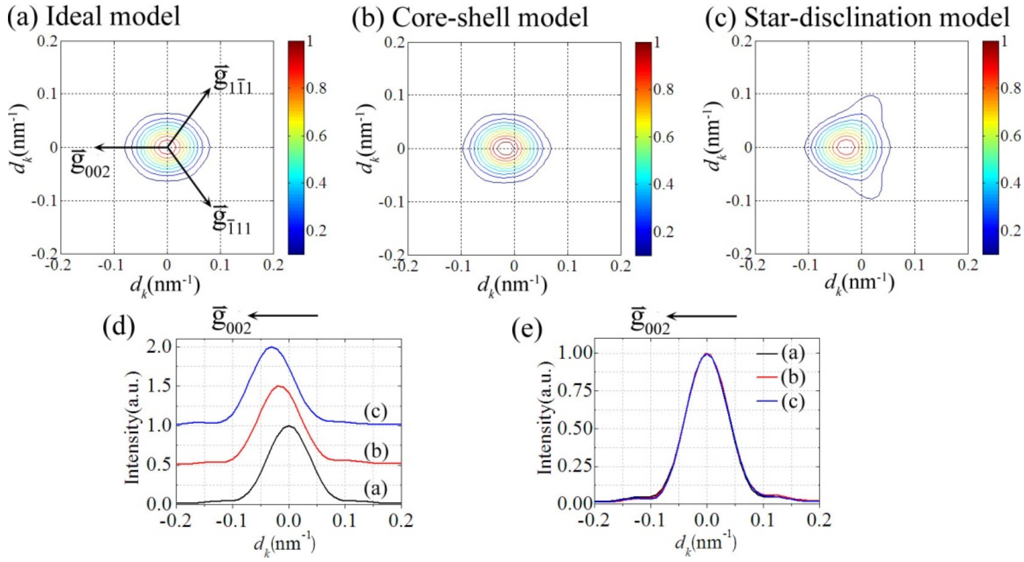

Figure S5: (a), (b) and (c) The contour map of (331) intensity distribution in the Ω plane kinematically simulated according to the ideal model (pure FCC segment), the core-shell model as shown in Fig. S3(c), and the star-disclination model, respectively. In (a), (b) and (c), the origin of the map is set at the (331) diffraction center of the ideal model, i.e. the strain-free pure FCC segment. (d) The $\vec{g}_{002}$ intensity profile comparison of the simulated (331) intensity map of the three structural models. (e) The curve shape comparison of the three $\vec{g}_{002}$ intensity profiles shown in (d) by aligning their intensity maximum together.

S5. The normal strain along $\vec{g}_{1\bar{1}1}$ and $\vec{g}_{\bar{1}11}$ direction about the star-disclination model

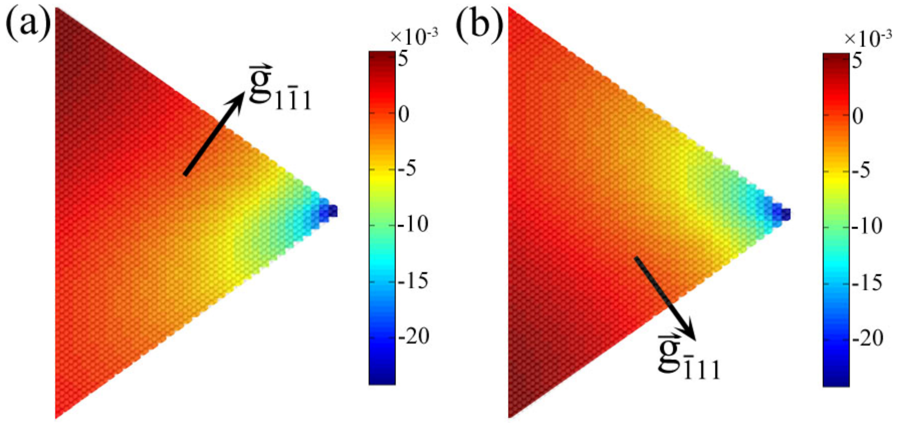

Figure S6: The map of the normal strain for the star-disclination model along the direction parallel to (a) $\vec{g}_{1\bar{1}1}$ and (b) $\vec{g}_{\bar{1}11}$, respectively.

S6. Round corner effect (shape effect)

The ideal model for Ag FTNWs exhibits a pentagonal cross-section bounded by five (001) surfaces. But some cross-sectional TEM observations have identified a morphology with round corners slightly deviated from the pentagonal cross-section.[1,4] Considering this situation, we have built a cross-sectional atomic configuration for a Ag single crystalline segment with round corners as shown in Fig. S7(b). In this configuration, the atomic lattice distortion is simulated by the star-disclination model. For comparison, the atomic structural model of a single crystalline segment for an ideal case of Ag pentagonal nanowire with star-disclination strain distribution has shown in Fig. S7(a). In Fig. S7(a) and (b) the viewing direction is along the nanowire axis. Based on the two atomic configurations shown in Fig. S7(a) and (b), the corresponding (331) intensity distribution simulated by the kinematic approximation is displayed at the right bottom corner, respectively. Directly viewing the simulation intensity map shown in Fig. S7(a) and (b), no evident difference can be found. Furthermore, the intensity profiles along the direction parallel to $\vec{g}_{002}$ and $\vec{g}_{1\bar{1}1}$ have been extracted and exhibited in Fig. S7(c) and (d), respectively. Here, we have not shown the intensity profile along $\vec{g}_{\bar{1}11}$ which is the same as the corresponding $\vec{g}_{1\bar{1}1}$ intensity profile due to the mirror symmetry for both structural models. In Fig. S7(c) and (d), the full width at half maximum (FWHM) for the round corner model is relatively narrow. But such diviation is too small in comparison with the instrumental broadening. Thus it cannot been identified in the current experimental examination. In addition, for $\vec{g}_{002}$ profile, the subsidiary peak intensity for round corner structural model is relatively weak, as indicated by arrow in Fig. S7(c). The intensity for this subsidiary peak is less than 5% of the maximum of (331) diffraction center as shown in Fig. S7(c). As the limitation of SNR (signal to noise ratio) for our currently used CCD detector, such weak subsidiary peaks cannot been detected. These comparison results

indicate that the round-corner cross sectional morphology has no evidently influence on the experimental (331) intensity distribution.

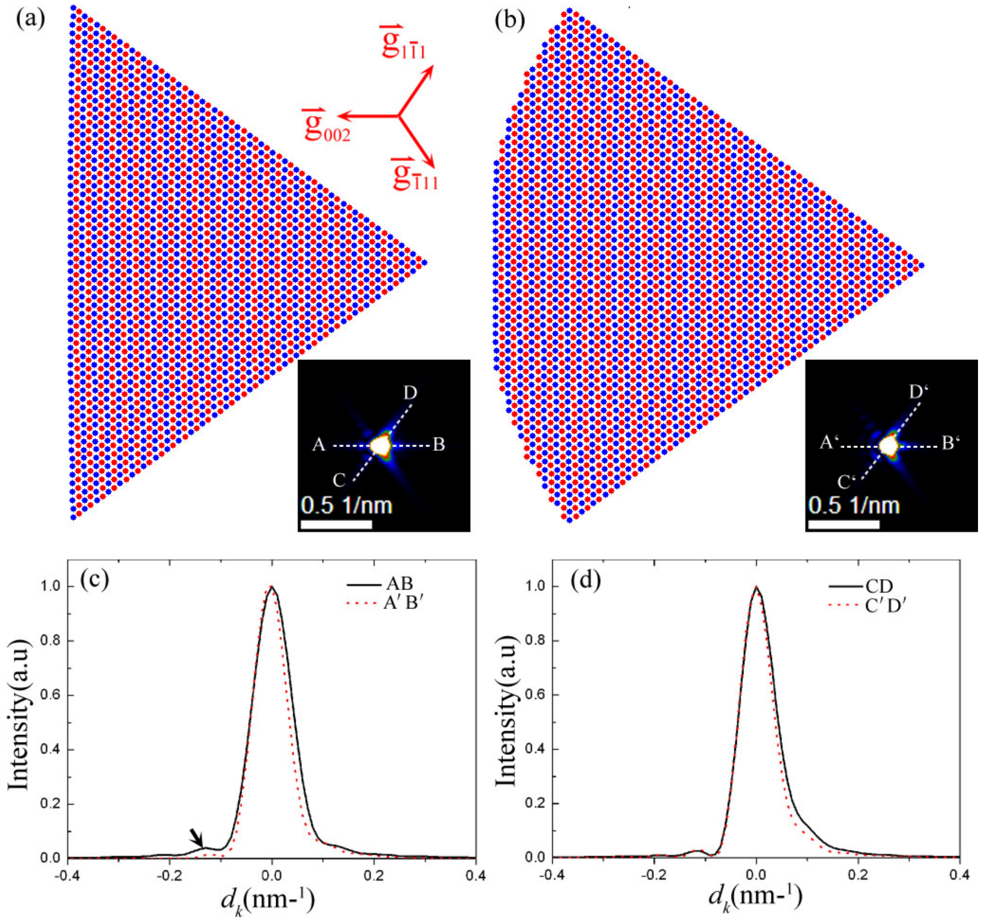

Figure S7: (a) The atomic structural model of a single crystalline segment for Ag pentagonal nanowire with star-disclination strain distribution and the corresponding simulated intensity distribution of (331) reflection. In (a), the directions of $\vec{g}_{002}$, $\vec{g}_{1\bar{1}1}$, $\vec{g}_{\bar{1}11}$ are demonstrated by red arrows. (b) The atomic structural model of a single crystalline segment for Ag FTNW with round corners. For (a) and (b), the viewing direction is along the [110] axis of the Ag FTNW. (c) The $\vec{g}_{002}$ intensity profiles along the lines indicated by AB in (a) and A'B' in (b), respectively. (d) The $\vec{g}_{1\bar{1}1}$ intensity profiles along the lines indicated by CD in (a) and C'D' in (b), respectively.

S7. Dynamic (Multiple Scattering) effect evaluation

Fig. S8(a) illustrates the diffraction geometry for the structural analysis of Ag FTNW with diameter of 30nm in this study. To evaluate the electron diffraction dynamic effect in our study case, here we suppose that the (331) diffracted beam when passing through the Ag nanowire is multiply scattered by assuming its transmitted intensity correlated with the projection thickness of the Ag nanowire [Fig. S8(b,c)]. In this simulation, we use the pure star-disclination model to build the atomic configuration of the Ag FTNW with diameter of 30nm schematically viewed in Fig. S4(b). According to the orientation relationship between the incident electron beam (EB) and the nanowire illustrated in Fig. S8(a), the simulated (331) intensity distribution of T1~T5 segment is shown in Fig. S8(d) and (e) which corresponding to the (331) beam intensity distribution below the nanowire demonstrated in Fig. S8(b) and (c), respectively.

The simulation results indicate that the multiple scattering of the electron beam affects the symmetric characteristics of (331) reflection diffracted from the single crystalline segments of T2~T5. Because that T1 is mirror-symmetric about the tilting axis, such symmetric feature is maintained by its (331) reflection without the influence by the electron dynamic effect. But T2~T5 segments are not in this case. Due to the dynamic effect, the mirror-symmetry about the direction parallel to $\vec{g}_{002}$ of the (331) reflection diffracted from T2 (or T3, T4, T5) has been breaking as shown in Fig. S8(d) and (e). Thus we can find the deviation in the magnitude between the integrated intensity ratios of $\vec{g}_{1\bar{1}1}$ and $\vec{g}_{\bar{1}11}$ line-profile for the single crystalline segment of T2 (or T3, T4, T5) as displayed in Fig. S8(f) and (g). The largest deviation (for T3 and T4) is only about 10% of the ratio (about 1.3, as indicated by dashed line in Fig. S8(f) and (g)) calculated by the kinematic approximation for the line-profile along $\vec{g}_{1\bar{1}1}$ (or $\vec{g}_{\bar{1}11}$) direction based on the pure star-disclination model. However, the experimental intensity ratios

shown in Fig. S8(h) are all less than the expected value for the pure star-disclination model in the kinematic approximation, and some ratios are even decreased by more than 20%.

Moreover, the dynamic calculation results shown in Fig. S8(d) and (e) also reveal that the intensity map containing five (331) reflections in the Ω plane exhibits a mirror-symmetric feature about the tilting axis indicated by the red dashed line. That reflects the real space symmetry between the electron beam and the Ag nanowire schematically shown in Fig. S8(a). As a result, such symmetric feature is also shown in the calculated integrated intensity ratios (I_2/I_1 as defined in the main text, Fig. 5) of the (331) intensity profile along the $\vec{g}_{1\bar{1}1}$ and $\vec{g}_{\bar{1}11}$ direction [Fig. S8(f) and (g)]. For T1 segment, the intensity ratios of the $(331)_{T1}$ profiles along $\vec{g}_{1\bar{1}1}$ and $\vec{g}_{\bar{1}11}$ direction are the same. But for T2 and T5, the ratio of $(331)_{T2}$ profile along $\vec{g}_{1\bar{1}1}$($\vec{g}_{\bar{1}11}$) direction is equal to that of the $(331)_{T5}$ intensity profile along $\vec{g}_{\bar{1}11}$($\vec{g}_{1\bar{1}1}$) direction. The similar result can also be found for T3 and T4 segment [Fig. S8(f) and (g)]. In contrast, the experimentally reconstructed (331) reflection of T1 segment has not show a mirror-symmetric feature as indicated by the intensity ratios shown in Fig. S8(h). Additionally, the experimental reflection pair of $(331)_{T2}$ and $(331)_{T5}$, as well as that of $(331)_{T3}$ and $(331)_{T4}$ totally do not show a mirror symmetry about the tilting axis [Fig. S8(h)]. Therefore, the evident discrepancies between the experimental result and the dynamic simulation suggest that the dynamic effect is not the most important factor inducing the experimental intensity-distribution divergences of (331) reflections diffracted from five single crystalline segments.

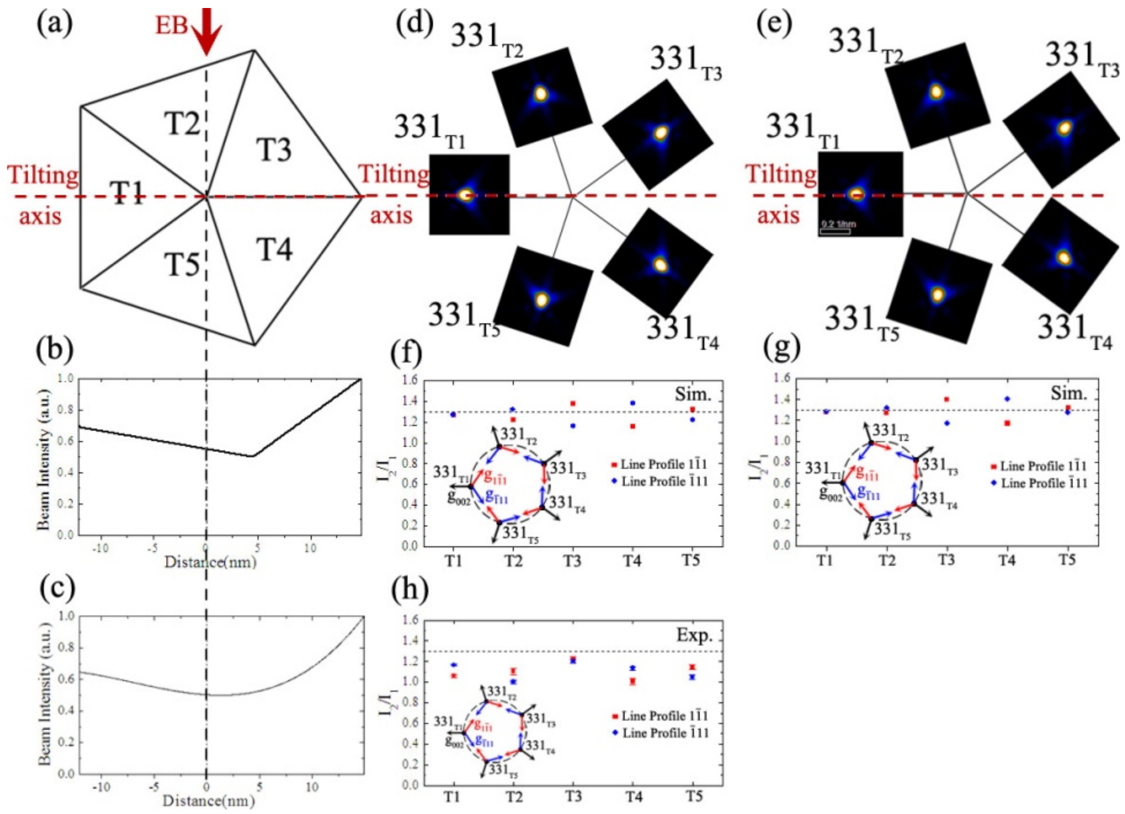

Figure S8: (a) The cross-sectional view of the diffraction geometry between the incident electron beam (EB) and the Ag FTNW with diameter of 30nm. The red dashed line indicates the direction parallel to the tilting axis. (b) and (c) illustrate the dynamic simulation assumption about the intensity distribution of (331) diffracted beam below the Ag FTNW as shown in (a). In (b) and (c), we assume a linear and a polynomial relationship, respectively, between the (331) beam intensity and the nanowire's project thickness. In this simulation the atomic configuration of single crystalline segment is built based on the pure star-dislclination model. (d) and (e) demonstrate the dynamic simulation result of (331) reflection intensity distribution in the Ω plane calculated according to the real-space (331) beam intensity distribution below the Ag nanowire shown in (b) and (c), respectively. (f) and (g) display the calculated integrated intensity ratios of the line-profiles along the characteristic direction of $\bar{g}_{1\bar{1}1}$ and $\bar{g}_{\bar{1}11}$, extracted from the simulated (331) intensity map shown in (d) and (e), respectively. (h) exhibits the integrated intensity ratios of the experimental line-profiles along $\bar{g}_{1\bar{1}1}$ and $\bar{g}_{\bar{1}11}$ direction, for the five single crystallites (T1~T5).

S8. Effect of the arragment of the stacking fault layers on the intensity fine structure

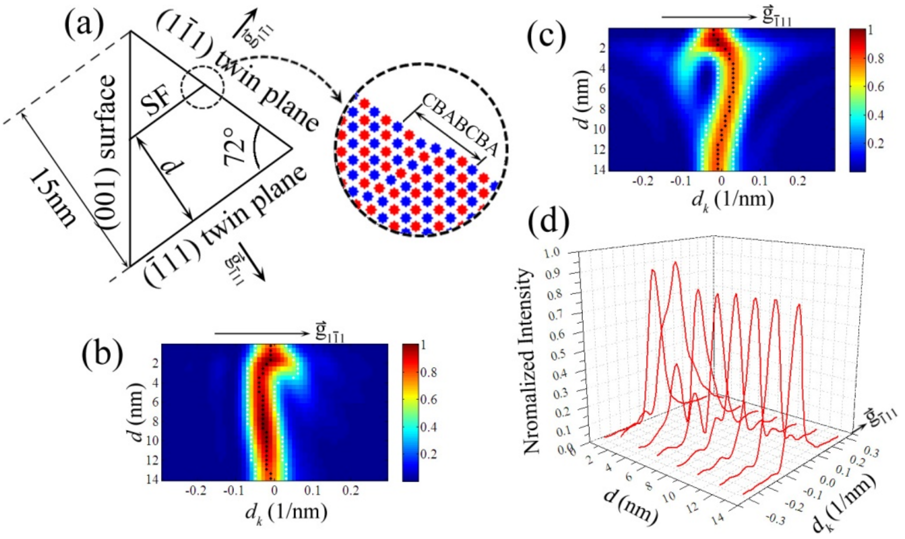

Figure S9: (a) Cross-sectional strucutural model of a Ag single crystalline segment with disclination core and a single layer of stacking fault parallel to ($\bar{1}11$) twin plane. The distance between the stacking fault layer and its parallel twin plane is indicated by *d*. According to the structural model shown in (a), the kinematically calculated contour color maps of (331) intensity line-profiles varying with *d*, along the direction of $\vec{g}_{1\bar{1}1}$ and $\vec{g}_{\bar{1}11}$ are demonstrated in (b) and (c), respectively. In (b) and (c), the black dots indicate the location of intensity maximum for each intensity line-profile. (d) The series of $\vec{g}_{\bar{1}11}$ line-profile curves aligning according to the distance of the stacking fault layer to its parallel ($\bar{1}11$) twin plane, i.e. *d* as indicated in (a). In (b)~(d), the origin for the deviation parameter (d_k) is set as the (331) diffraction center for the ideal Ag segment model without lattice distortion and defects.

On the basis of star-disclination atomic configuration for the single crystalline segment, we introduce one atomic layer of stacking fault parallel to a {111} twin plane of the segment. This operation changes the regular close packing, ···ABCABC···, to the packing sequence of ···ABCBABC···, schematically displayed in Fig.S9(a). According to this atomic configuration, we have simulated a series of 2D intensity distribution map for (331) reflection by varying the distance between the stacking fault layer and the parallel twin plane (indicated

as *d* in Fig.S9(a)). In Fig.S9(b-c), we present the color map of (331) intensity line-profile varying with the distance between the stacking fault layer and the twin plane. The original point for the deviation vectors is set as the (331) diffraction center for the ideal Ag segment model without lattice distortion and defects [Fig. 4(a)]. The simulated results shown in Fig.S9(b-c), clearly indicate that the intensity fine structure variation due to the introduction of stacking fault strongly depends on the location of the stacking fault layer.

Kinematic simulation for the pure star-disclination model evidently shows that the intensity streaking along $\vec{g}_{\bar{1}11}$ direction is stronger than that along the opposite direction (i.e. $\vec{g}_{1\bar{1}\bar{1}}$) [Fig.5(b)]. Likewise, the intensity profile along $\vec{g}_{1\bar{1}1}$ also show such a feature [Fig.5(a)]. Fig.S9(b) proves that such asymmetric feature can not been changed, but even can be enhanced along the direction perpendicular to one twinn plane, while a single layer of stacking fault paralled to the other twin plane is incorperated into the single crystallite.

The most evident change of the intensity fine structure due to the introduction of stacking fault layer occurs along the direction perpendicular to the stacking faul plane. As shown in Fig.S9(c), the presence of single layer of stakcing fault parallel to ($\bar{1}11$) plane could induce apparent streaking, even splitting of the (331) diffraction spot along $\vec{g}_{\bar{1}11}$ direction. The (331) diffraction spot splitting can be observed, while the distance of ($\bar{1}11$) stacking faul layer to its parallel twin place is in the range from about 2nm to 6nm. As the distance is in the range from about 6nm to 10nm, the intensity streaking of (331) reflection towards $\vec{g}_{1\bar{1}\bar{1}}$ direction is more evident with respect to the opposite direction. But in the case that the stacking fault layer is close to the triangular corner (i.e. d > 10nm) as shown in Fig. S9(c, d), the (331) reflection also exhibits more intensive flare in the $\vec{g}_{\bar{1}11}$ direction with respect to the opposite direction, similarly to the pure star-disclination model but with the diference that a weak subsidiary peak possibly apears along the $\vec{g}_{\bar{1}11}$ direction. In Fig. S9(b, c) it also can been found that if the stacking fault layer is next to its parallel twin plane (d<2nm), the intensity

line-profiles along the characteristic direction of $\vec{g}_{1\bar{1}1}$ or $\vec{g}_{\bar{1}11}$ also do not exhibit evident variation in the fine strucutre in comparing with the pure star-disclination model.

S9. Cross-section area estimation

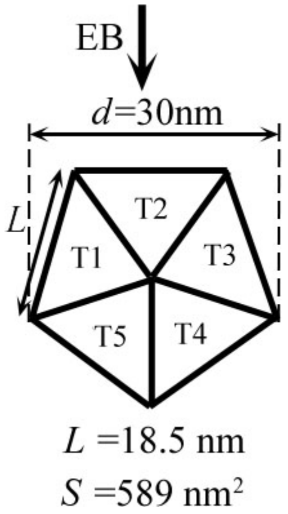

Figure S10: The estimation result about the cross-section area of the Ag FTNW under study. The 30nm diameter of the nanowire is measured from Fig. 2(a). In Fig. 2(a) the nanowire shows a mirror-symmetric contrast about the long axis which corresponding to the symmetric orientation of the nanowire's cross-section with respect to the incident electron beam (EB) as shown in this figure.

References

1. Sun, Y., *et al.* Ambient-stable tetragonal phase in silver nanostructures. *Nat. Commun.* **3**, 971 (2012).
2. De Wit, R. Patial disclinations. *J. Phys. C: Solid State Physics* **5**, 529-534 (1972).
3. McCarthy, E. K., Bellew, A. T., Sader, J. E. & Boland, J. J. Poisson's ratio of individual metal nanowires. *Nat. Commun.* **5**, 4336 (2014)
4. Niekiel, F., Bitzek, E. & Spiecker, E. Combining atomistic simulation and x-ray diffraction for the characterization of nanostructures: a case study on fivefold twinned nanowires. *ACS Nano* **8**, 1629-1638 (2014).
